# Biological nitrogen fixation and prospects for ecological intensification in cereal-based cropping systems

**DOI:** 10.1016/j.fcr.2022.108541

**Published:** 2022-07-01

**Authors:** Jagdish K. Ladha, Mark B. Peoples, Pallavolu M. Reddy, Jatish C. Biswas, Alan Bennett, Mangi L. Jat, Timothy J. Krupnik

**Affiliations:** a Department of Plant Sciences, University of California, Davis, CA, USA; b Commonwealth Scientific and Industrial Research Organisation, Canberra, Australia; c The Energy and Resources Institute, New Delhi, India; d Krishi Gobeshona Foundation, Dhaka, Bangladesh; e International Maize and Wheat Improvement Center, New Delhi, India; f International Maize and Wheat Improvement Center, Dhaka, Bangladesh

**Keywords:** Nitrogen cycle, Symbiotic nitrogen fixation, Non-symbiotic nitrogen fixation, Legumes, Diazotrophs, Crop nitrogen nutrition

## Abstract

The demand for nitrogen (N) for crop production increased rapidly from the middle of the twentieth century and is predicted to at least double by 2050 to satisfy the on-going improvements in productivity of major food crops such as wheat, rice and maize that underpin the staple diet of most of the world’s population. The increased demand will need to be fulfilled by the two main sources of N supply – biological nitrogen (gas) (N_2_) fixation (BNF) and fertilizer N supplied through the Haber-Bosch processes. BNF provides many functional benefits for agroecosystems. It is a vital mechanism for replenishing the reservoirs of soil organic N and improving the availability of soil N to support crop growth while also assisting in efforts to lower negative environmental externalities than fertilizer N. In cereal-based cropping systems, legumes in symbiosis with rhizobia contribute the largest BNF input; however, diazotrophs involved in non-symbiotic associations with plants or present as free-living N_2_-fixers are ubiquitous and also provide an additional source of fixed N. This review presents the current knowledge of BNF by free-living, non-symbiotic and symbiotic diazotrophs in the global N cycle, examines global and regional estimates of contributions of BNF, and discusses possible strategies to enhance BNF for the prospective benefit of cereal N nutrition. We conclude by considering the challenges of introducing *in planta* BNF into cereals and reflect on the potential for BNF in both conventional and alternative crop management systems to encourage the ecological intensification of cereal and legume production.

## Highlights

• Biological N fixation (BNF) is the single largest global input of reactive nitrogen.
• Critical review of current knowledge of BNF in cereal-based cropping systems.
• Global and regional estimates of the nutrient supply contribution of BNF.
• Discussion of the challenges associated with introducing *in planta* BNF into cereals.
• Overview of opportunities to enhance use of BNF in ecological intensification.

## 1 Introduction

Nitrogen (N) is unique among the major soil nutrients in that it originates from the atmosphere, and its transformations and movement in an ecosystem are mediated almost entirely by the water cycle and biological processes. The atmosphere contains a large, well-mixed but biologically unavailable pool of di-nitrogen (N_2_) gas (4 × 10^9^ Tg N), of which a relatively small part (473 Tg N) was calculated by Fowler et al. (2015) as being converted annually to biologically available or reactive pools of N. These transformations were estimated to be mediated through (a) a natural non-biological process (lightning 5 Tg N), (b) biological N_2_ fixation (BNF: 128 Tg N for natural terrestrial ecosystems, 120 Tg N for marine and aquatic ecosystems and 60 Tg N in agricultural ecosystems), (c) industrial fixation of ammonia (NH_3_) via the Haber-Bosch process (120 Tg N), and (d) fossil fuel combustion (40 Tg N). Of the total 473 Tg N fixed from atmospheric N_2_, 220 Tg N was deemed to be contributed by anthropogenic activities (Fowler et al., 2015).

The biological fixation of atmospheric N_2_ is an essential process in the biosphere, second in importance only to photosynthesis for the maintenance of life on earth (Stevenson, 1982). Only a few key genera of prokaryotic organisms that contain the genetic information needed to synthesize the enzyme nitrogenase possess the ability to convert gaseous N_2_ into NH_3_ which can then be biochemically modified to generate different organic forms of N (Pate, 1980, Giller, 2001; Table 1). While nitrogenase catalyzes the conversion of N_2_ to NH_3_ under normal temperature and atmospheric pressure, the industrial production of fertilizer N relying upon the Haber-Bosch process requires high temperature and pressure to enable the same reaction (Smil, 2001, Erismann et al., 2008). Prior to the wide-spread availability of N fertilizers manufactured using the Haber-Bosch process, BNF was the primary source of reactive N used in agriculture (Smil, 2001). Legumes grown for grain or forage were identified as the key agents responsible for the N inputs more than 100 years ago (Hellriegel, 1886), although it is now recognized that the free-living and non-symbiotic N_2_ fixation by a diverse range of aerobic and anaerobic organisms using a wide variety of substrates (e.g., soil and organic material, including crop residues; Table 1) also play a contributing role (Ladha et al., 2016). However, since the early 1960s the progressive increase in applications of fertilizer by farmers has seen synthetic N become the more dominant source of N input for food production (Smil, 2001, Crews and Peoples, 2004).

**Table 1:** Key genera of free-living and symbiotic diazotrophs of agricultural significance and examples of associated host plant species.

Heterotrophic		Phototrophic	
Free-living	Symbiotic (host)	Free-living	Symbiotic (host)
**Aerobic**	*Azorhizobium*	**Cyanobacteria**	Anabaena (Azolla)
*Azotobacter*	(sesbania)	*Gloeocapsa*	
*Azospirillum*	*Bradyrhizobium*	*Trichodesmium*	
*Azoarcus*	(peanut, soybean, vigna)	*Nostoc*	
*Beijerinckia*	*Mesorhizobium*	*Anabaena*	
*Rhizobium*	(chickpea, milk vetch)	*Calothrix*	
**Facultative anaerobic**	*Rhizobium*	**Photosynthetic bacteria**	
*Bacillus*	(common bean, peas, lentils, vetch, medicago, sesbania)	*Rhodospirillum*	
*Klebsiella*	*Sinorhizobium*	*Rhodopseudomonas*	
*Enterobacter*	(soybean, sesbania)	*Chromatium*	
*Erwinia*		*Chlorobium*	
**Anaerobic**			
*Clostridium*			
*Desulfobirio*			

While some soils can mineralize sufficient N to satisfy the growth requirements of high yielding crops (e.g. 300–400 kg mineral N ha^-1^; Peoples et al., 2004a; Angus et al., 2015), additional reactive N produced biologically or chemically is generally also needed to support agricultural productivity. Unfortunately, not all the reactive N pool is fully recovered by food crops; a portion is frequently lost, resulting in serious environmental and human health consequences (Peoples et al., 2004b, Galloway et al., 2008, Schlensinger, 2009, Vitousek et al., 2009, Canfield et al., 2010, Ladha et al., 2020). In the USA, for example, Sobota et al. (2015) calculated the potential annual environmental damage and health implications resulting from N lost from synthetic N to be approximately USD210 billion (range: USD81–441 billion yr^−1^). This has led some to suggest that humans have transgressed the sustainable planetary ‘boundary' (or limit) for sustainable N management (Erismann et al., 2008, Steffen et al., 2015, Rockström, 2015).

Synthetic fertilizer N is a major production expenditure for farmers globally. For this reason, many governments utilize taxpayer funds or other mechanisms to subsidize (and thus encourage) the use of N fertilizer. For example, during 2020–2021 the Government of India provided a cash subsidy equivalent to around USD10 billion for this purpose (Government of India, 2021). There is a high capital commitment associated with the construction of N fertilizer manufacturing facilities, which has been a constraint to expanding synthetic fertilizer production, especially in developing countries (Good, 2021).

Wheat (*Triticum aestivum*), rice (*Oryza sativa*) and maize (*Zea mays*) are globally the most important and widely grown food crops, providing the bulk of the calories and proteins consumed by humans, either directly as grain or indirectly through livestock products. The combined area of arable land occupied by these three cereals in 2019 represented 575.4 million hectares (Mha), producing 2663 million tonnes (Tg) of grain (Table 2; FAOSTAT, 2021). This grain underpins the staple diet of billions of households in both the developing and developed world. The four-fold improvement in cereal production that has been achieved since 1961 has been accompanied by an almost nine-fold increase in fertilizer N use (Smil, 2001, Erismann et al., 2008, FAOSTAT, 2021). Of the total 102.5 Tg of synthetic N produced by the Haber-Bosch process and applied globally in the production of annual and perennial crops in 2014/15, some 52.6 Tg N (51.2% of N supplied to all crops) was applied to wheat (18.2%), rice (15.2%) and maize (17.8%; Heffer et al., 2017). Just three countries were responsible for consuming more than half of this fertilizer N (China – 22.7%; India – 18.5% and USA – 14%; Table 3). The European Union (EU) was the next highest user of fertilizer N for cereal production (9.2%), with a further four countries in Asia and South America applying more than 1 Tg of fertilizer N to either wheat, rice or maize (Table 3).

**Table 2:** Comparison of land area (million ha, or Mha) sown to major cereals or grain legumes in different geographic regions, and the percentage of the total area used for cereal and legume production under grain legumes in 2019^a^.

Region	Wheat	Rice	Maize	Total 3 cereals	Legume oilseeds^b^	Pulse legumes^c^	Total grain legumes	% Area legumes^d^
Americas	34.6	5.7	71.7	112.0	92.7	11.6	104.3	
South America	9.3	4.1	28.2	41.6	59.5	3.9	63.4	60
North America	24.7	1.0	34.5	60.2	33.2	4.7	37.8	39
Asia	98.6	138.6	66.5	303.7	32.1	43.7	75.8	
South Asia	49.4	61.4	12.2	123.0	16.1	33.6	49.7	29
East Asia	24.3	32.7	41.9	98.9	13.3	3.1	16.4	14
South-East Asia	0.06	43.9	11.2	55.2	2.5	5.1	7.6	12
West Asia	10.7	0.2	0.9	11.8	0.1	1.3	1.4	11
Central Asia	14.1	0.4	0.3	14.8	0.1	0.6	0.7	5
Africa	9.7	17.1	41.2	68.0	19.6	27.3	46.9	41
Europe	62.3	0.6	18.3	81.2	5.6	4.9	10.5	11
Oceania/Australia	10.4	0.02	0.1	10.5	0.05	3.4	3.5	25
World (Mha)	215.6	162.0	197.8	575.4	150.1	90.9	240.6	

a Data obtained from FAOSTAT (2021) and presented in declining regional areas sown to grain legumes.

b Soybean and groundnut.

c All other legume crops grown for dry grain.

d % area under legume crops = 100× (area total grain legumes)/ (area total grain legumes)+ (area of wheat+rice+maize).

**Table 3:** Individual countries or geographic region where more than 1Tg of fertilizer N was applied to either wheat, rice or maize in 2014/15. Data in parentheses indicate the combined amounts of fertilizer N supplied as a percentage of the global total.

Region or Country	Amount of fertilizer N applied per crop (Tg N)			Fertilizer N applied to all 3 cereals	% Global total N applied to all 3 cereals
	Wheat	Rice	Maize	(Tg N)	(%)
China	3.40	3.90	4.65	11.95	(22.7)
India	3.95	4.93	0.83	9.71	(18.5)
USA	1.57	0.20	5.58	7.35	(14.0)
European Union	3.28	0.05	1.51	4.84	(9.2)
Pakistan	1.36	0.50	0.15	2.01	(3.8)
Indonesia	0	1.19	0.48	1.67	(3.2)
Brazil	0.18	0.18	1.06	1.42	(2.7)
Bangladesh	0.02	1.03	0.03	1.08	(2.0)
Sub-total	13.8	12.0	14.3	40.0	(76.1)
% global total applied per crop	(73.6)	(76.8)	(78.1)		
Global total N applied (Tg N)	18.7	15.6	18.3	52.6	(100)

^a^Data obtained from Heffer et al. (2017) and presented in order of declining fertilizer consumption.

Based on current farming practices, studies suggest that the demand for synthetic N is likely to double or triple to support the on-going improvements in crop productivity that will be necessary to meet the anticipated dietary demands of a population of 9.7 billion people by the middle of the century (Erismann et al., 2008, Rivas and Nonhebel, 2017, FAO, 2018). There are still large areas in the world where the supply of N is insufficient to achieve food and nutritional security. Sub-Saharan countries in Africa, for example, account for only 1.5% of world fertilizer consumption, applied at an average rate of just 13 kg N ha^–1^, and other options are clearly desperately needed (Franke et al., 2018). This challenging situation is not expected to change dramatically in the future without major agricultural and market policy interventions (Alexandratos and Bruinsma, 2012, Pradhan et al., 2015, Ciceri and Allanore, 2019, Ladha et al., 2020).

Despite the adoption of relatively good management practices for synthetic N by many cereal-growers, N use efficiency (NUE) (measured as kg plant N harvested per kg synthetic fertilizer N applied) is frequently less than 50% (Ladha et al., 2005). Analyses indicate that NUE has either stagnated or declined over time in most countries except the USA and Europe (Zhang et al., 2015a, Ladha et al., 2020). While poorly timed application of synthetic N results in a mismatch between flushes of N supply and crop N demand which greatly increases the susceptibility of the surplus fertilizer N to loss processes, the slower availability of N derived from BNF sources such as legumes might be expected to better synchronize supply with demand, as the rate of N release from organic residues and the N requirements for crop growth are both regulated by available water and temperature (Crews and Peoples, 2005, Ladha et al., 2020). Certainly, this proposition is supported by numerous studies that have demonstrated lower losses of the N derived from legume systems than N fertilized cropping and intensive forage systems (Peoples et al., 2004b, Crews and Peoples, 2005, Jensen et al., 2012, Jeuffroy et al., 2013, Schwenke et al., 2015, Costa et al., 2021). However, it should also be acknowledged that in some climates, soil types and farming systems can be an elevated risk of leaching or volatile losses of N mineralized from the N-rich legume residues following the end of the growing season (Fillery, 2001, Watson et al., 2017, Williams et al., 2017), although often there are opportunities for this to be managed with the use of cover crops (Kaye and Quemada, 2017, Plaza-Bonilla et al., 2017, Watson et al., 2017). The added advantage of N being supplied from the organic residues of N_2_-fixing food or fodder legumes substantially lower fossil energy C costs associated with their production than N fertilized systems (Erismann et al., 2008, Jensen et al., 2020, Costa et al., 2021).

Considering that the demand for synthetic N is expected to continue to grow to meet the nutritional requirements for future increases in cereal production, and the environmental and human health problems linked to N lost from agriculture are likely to intensify, we suggest that it is timely to reassess the capacity for biological sources of N to augment N supplied by fertilizer.

In this paper we discuss the ecological services provided by free-living, non-symbiotic and plant symbiotic diazotrophs and the contribution of BNF to the global N cycle and in agroecosystems, with a focus on cereal-based cropping systems. We also review global and regional estimates of non-symbiotic and symbiotic BNF currently occurring in wheat-, rice- and maize-based cropping systems, evaluate the prospects for increasing inputs of BNF from these sources, and examine progress in research efforts aimed at transferring BNF capabilities to cereals to enable them to fix their own N.

## 2 The N cycle and essential role of BNF

Nitrogen fixation is the key process in the global N cycle. Dinitrogen is introduced into the biosphere as chemically triple bonded N≡N. Almost 10^3^ kJ mol^-1^ are required to break the triple bond and convert it to NH_3,_ regardless of whether this occurs via BNF or the Haber-Bosch process. In the case of BNF, the NH_3_ formed by nitrogenase activity is rapidly converted to ammonium (NH_4_^+^) which is subsequently utilized for the biochemical synthesis of amino acids and proteins which are temporarily stored in microbial or plant biomass (Pate, 1980). Upon senescence and death, these organic sources of N add to the soil organic N (SON) pool, a portion of which will subsequently be mineralized by soil microbes and released as an available form of N, such as nitrate (NO_3__-_), that can be assimilated by other members of the soil microbial community and plant roots.

Unlike other elements, N is not completely recycled by organisms. Instead, it is continually lost via different loss pathways including: (a) microbially-mediated denitrification of NO_3_^-^ under anaerobic conditions resulting in the emissions of potent greenhouse gases such as nitrous oxide (N_2_O) from the soil, (b) NH_3_ volatilization from animal urine patches, compost and alkaline soils, (c) NO_3_^-^ leached beyond the crop rooting zone to groundwater, and (d) organic and inorganic forms of N lost in run-off water and erosion (Stevenson, 1982, Fillery, 2001, Ledgard, 2001, Peoples et al., 2004b, Galloway et al., 2008, Lenhart et al., 2015, Timilsina et al., 2020). BNF can therefore be an important mechanism for replenishing the reservoirs of organic N in a plant-soil system. Soil acts both as an N sink (gain) and an N source (loss) and is in dynamic flux. The ability of a soil system to store N lies in the balance between net gains and net losses. Consequently, BNF and the plethora of N loss pathways can be considered to be the basic processes underlying the N-cycle (Stevenson, 1982).

## 3 Nitrogen-fixing organisms

Only prokaryotic organisms possess the ability to fix atmospheric N_2_. This capacity is restricted to a relatively small but diverse group of bacteria and blue-green algae (collectively referred to as diazotrophs) which belong to the kingdoms eubacteria and archaebacteria (Table 1). Although a relatively limited number of bacterial species fix N_2_, they represent a wide variety of phylogenetically and physiologically distinct types which occupy different ecological niches (Eady, 1991). The diazotrophs can be heterotrophs or phototrophs. Heterotrophs can grow in the dark and rely on a supply of reduced carbon (C) such as sugars or organic acids either released into the plant root rhizosphere (Vives-Peris et al., 2019) or derived from materials such as plant residues (Roper and Ladha, 1995). Phototrophs on the other hand use light as a source of energy to reduce CO_2_ and include N_2_-fixing systems such as cyanobacteria (blue-green algae) in floodwater and the soil–water interface of lowland rice (Roger and Ladha, 1992). Both heterotrophs and phototrophs function in nature as free-living organisms, and in non-symbiotic associations or symbiotic relationships with living plants (Sprent and Sprent, 1990, Giller, 2001). In symbiotic N_2_ fixation, diazotrophic symbionts (e.g., *Rhizobium* spp., and *Anabaena azollae*; Table 1) reside within specific structures provided by their plant hosts (legume root nodules or specialized cavities in the upper surface of the aquatic fern *Azolla* leaves; Giller, 2001). While free-living diazotrophs tend to have no or little specificity when they associate with a plant, a symbiotic association generally has a very high degree of specificity between a diazotroph and its host. Nevertheless, the question as to what extent symbiotic N_2_-fixing plants are facultative or obligate fixers remains unresolved (Sheffer et al., 2015).

### 3.1 Free-living and non-symbiotic diazotrophs

Free-living diazotrophs can occur in diverse habitats (e.g., soil, water, organic material, including crop residue or on living plants). When they partner with non-leguminous plants including cereals, either in free-living conditions or in association with plants, they are defined as being non-symbiotic. They primarily inhabit the soil–root interspace but can sometimes also enter and survive inside plant tissues (e.g., roots and/or stems). In this case, they are referred to as endophytes.

The plant rhizosphere favors nitrogen fixation because of a relatively more conducive environment with high availability of C substrates and low oxygen (O_2_) partial pressure. Bacteria grow and fix N_2_ in the rhizosphere (soil surrounding the roots), rhizoplane (outer surface of roots) and histosphere (inside the roots). Rhizoplane bacteria are those that are detached from the roots by vigorous shaking; histosphere bacteria are those obtained after macerating the root samples from which rhizoplane bacteria have been removed (Watanabe et al., 1981). Baldani and Dobereiner (1980) considered histosphere (or endorhizosphere) bacteria to be those that remain alive after the root had been ‘sterilized’ by chloramine. Nevertheless, the boundary between associative or free-living and endophytic bacteria is not very clear. The mode of entry of histospheric or endophytic diazotrophs is largely through cracks created at the emergence points of lateral roots. They establish primarily in intercellular spaces in cortex and the xylem vessels (James, 2000). Sometimes endophytes are referred to as endosymbionts, but in the true sense they are not, even though they invade plant tissues. Unlike endosymbiotic association with legumes, the presence of endophytes does not initiate the plant to generate specialized or differentiated structures such as nodules to host the micro-organism. Also, there is no evidence that bacteria truly colonize the root intercellularly in non-leguminous plants. The growth of bacteria inside the plant appears to be limited by the plant’s own defense systems (Rosenblueth and Martinez-Romero, 2006). Moreover, is likely that the observed intercellular colonization of root cells occurs in the older parts of roots, where the cells are generally damaged (James, 2000).

Most free-living soil diazotrophs such as *Azospirillum, Herbaspirillum, Azotobacter, Azoarcus, Pseudomonas, Klebisella, Enterobacters, Burkolderia* and *Gluconobacter* have been found in the rhizosphere and as endophytes in wheat, rice, maize and other plants (James, 2000, Kennedy and Islam, 2001, Eskin et al., 2014, Ladha and Reddy, 2019). However, a diverse community of diazotrophs that could fix N_2_ (including: *Pseudomonas fluorescens, Klebsiella pneumoniae, Azospirillum* sp*., Azospirillum lipoferum, Enterobacter cloacae, Derxia gummosa, Xanthobacter* and *Flavobacterium*) have also been isolated from both the rhizosphere and in mucigel on adventitious roots above the soil surface of a Mexican indigenous maize landrace from southern Mexico (Gonzalez-Ramirez and Ferrera-Cerrato, 1995). Subsequent 16S rRNA gene and shotgun metagenome sequencing has confirmed the high level of diversity of the diazotrophic microbiota associated with underground and aerial roots, stems, and aerial root mucilage of maize landraces (Van Deynze et al., 2018).

Normally, heterotrophic free-living diazotrophs are active, fixing N_2_ in surroundings rich in organic C and low in N. Diverse diazotrophs can be obligate aerobes, or facultative or obligate anaerobes (Table 1). Excepting *Azotobacter*, N_2_ fixation occurs only under anaerobic or micro-aerobic conditions. *Azotobacter* is a strict aerobe capable of metabolizing and fixing N_2_ in an aerobic environment. Photosynthetic bacteria are largely phototrophic and fix N_2_ strictly anaerobically, which limits their habitats. The other key group of free-living phototrophs are cyanobacteria with the ability to fix N_2_ under aerobic conditions. These are largely aquatic in nature and are important in flooded rice ecosystems (Watanabe and Cholitkul, 1979) but can also occur as biocrusts on the soil surface in rainfed wheat fields (Witty et al., 1979). In filamentous cyanobacteria, BNF takes place in thick-walled cells called heterocysts which lack photosystem II (the absence of O_2_ evolution) thereby protecting the O_2_ sensitive nitrogenase enzyme (Wolk, 1996). On the other hand, the plant’s vegetative cells photosynthesize and evolve O_2_. Conversely, unicellular cyanobacteria, such as *Cyanothece*, are capable of N_2_ fixation but have no heterocysts developed possessing a unique biphasic mechanism in which oxygenic photosynthesis occurs during the day and N_2 is fixed_ only at night (Heimann and Cirés, 2015).

Rice paddies represent a flooded soil ecosystem with unique diversity, structure, and dynamics of microbial communities. Flooding causes rapid depletion of O_2_ in the submerged soil, leading to the establishment of macro- and micro-environments (oxic surface, anoxic bulk and rhizosphere) differing in redox state (Liesack et al., 2000, Ladha and Reddy, 2019). Flooded rice is a “microbial Babylon” with a variety of microorganisms (Stewart et al., 1979). A wide range of diazotrophs, including cyanobacteria, have been reported in diverse rice ecosystems (Supplementary Material Table S1): (1) photosynthetic cyanobacteria: heterocystous, non-heterocystous, unicellular (Chrococcaceans) and pleurocapsalean forms; (2) heterotrophic aerobes*:* sulfur-oxidizing and methane-oxidizing; (3) heterotrophic facultative anaerobes; (4) heterotrophic obligate anaerobes and sulfate-reducing; and (5) photosynthetic microbes (Watanabe and Cholitkul, 1979, Ladha et al., 1983, Barraquio et al., 1983). Barraquio et al. (1997) and Stoltzfus et al. (1997) isolated a large diversity of diazotrophic (up to 10%) and non-diazotrophic bacteria from rice tissues, some of which were capable of recolonization when re-inoculated on to sterile rice seedlings. These microbes reside along an O_2_ concentration gradient around rice roots. Rice roots also provide a steady supply of energy-rich organic compounds (through rhizodeposition which includes sloughed off cells as well as C leakage) that favors BNF (Vives-Peris et al., 2019). Studies based on molecular phylogeny of the DNA sequences generated by PCR amplification of N_2_-fixing genes also confirmed the existence of a broad range of diazotrophs in rice (Ueda et al., 1995, Reinhold-Hurek and Hurek, 2011, Edwards et al., 2015).

Because of their enormously diverse characteristics of metabolism and habitat, free-living diazotrophs are of great interest, not only ecologically but also agronomically. Many free-living diazotrophs have also been found to exhibit functions other than N_2_ fixation which may include (a) plant growth promotion, (b) mobilization of minerals and nutrient acquisition, (c) stress tolerance, (d) defense against pathogens, and (e) bioremediation. Such effects unrelated to BNF have previously been extensively reported (Kennedy et al., 2004, Barea et al., 2005, Doty, 2011, Gaiero et al., 2013, Mendes et al., 2013, Maseko et al., 2020) and will not be considered further here.

### 3.2 Diazotrophs in symbiosis with plants

Diazotrophic eubacteria are also found in symbiotic associations with plants and establish active N_2_ fixation. When diazotrophs are in symbiosis with plants, they are referred as symbionts. Both heterotrophic and phototrophic diazotrophs establish N_2_-fixing symbioses – the former are rhizobia, which associate with plant family Leguminosae (or Fabaceae; commonly known as legumes: Mylona et al., 1995; Hirsch et al., 2001; Berrada and Fikri-Benbrahim, 2014) and the latter are cyanobacteria that associate with *Azolla* (Becking, 1987, Peters and Meeks, 1989). The Leguminosae family is the third largest family of flowering plants, consisting of over 20,000 species (Stagnari et al., 2017). Legumes are grown agriculturally, primarily for human consumption and for livestock forage but are also used as a source of N-rich green manure to enhance soil N availability. Given its aquatic nature, *Azolla* is used only in flooded lowland rice systems solely as a green manure (Roger and Watanabe, 1986, Peoples and Craswell, 1992, Giller, 2001). There are other diazotroph-plant symbioses (Sprent et al., 1987), but they play no significant role in cereal-based farming systems and will not be included in the following discussion.

#### 3.2.1 Grain legumes

Table 4 lists some of the most widely grown grain legumes included in cereal-based farming systems. Globally, legume oilseeds (soybean and groundnut) and pulse crops (grown for dried grain) occupied 240.6 Mha in 2019 which was equivalent to 42% of the combined land area sown by wheat, maize and rice (Table 2). The largest areas of grain legumes occurred in the Americas (104.3 Mha) with soybean and pulses being grown on 60% of the total land under legumes, wheat, rice or maize in South America and 39% in North America (Table 2). Asia had the next largest area of grain legumes (75.8 Mha) of which > 65% was in South Asia, reflecting 29% of the land under legume and cereal cropping dedicated to soybean, groundnut and pulses. By comparison, other regions in Asia grew grain legumes on just 5–14% of the land under legume and cereal cropping (Table 2). Also in 2019, Africa was another region with large areas of grain legumes and a high proportion of the cropping land growing legumes (46.9 Mha and 41%, respectively), while Europe had one of lowest (10.5 Mha and 11%, respectively; Table 2) covering only 1.6% of arable land (Watson et al., 2017). However, there are major differences in the use of grain legumes by individual European countries, ranging from 1% or less of the arable land in Denmark, Germany and the Netherlands (only 1–2% of cropping sequences contain pulses) to 5–7% of arable land in Italy and Lithuania (13–17% of cropping sequences contain pulses; Stoddard, 2017; Watson et al., 2017).

**Table 4:** Examples of grain legumes grown in cereal-based cropping systems.

Species	Common name (s)	Principal regions of production and comments
*Arachis hypogaea*	groundnut, peanut	West and South Asia, Africa, North and South America
*Cajanus cajan*	pigeon pea	South Asia, Africa
*Cicer arietinum*	chickpea, Bengal gram	Mainly South Asia, but widely grown elsewhere
*Glycine max*	soy(a)bean	North and South America, East and South Asia
*Lablab purpureus*	lablab bean, lablab, hyacinth bean	Native to Africa, but also cultivated throughout tropical regions of Asia
*Lathyrus sativus*	grasspea, blue sweet pea, chickling pea	South Asia and East Africa, particularly in areas prone to drought
*Lens culinaris*	lentil	North America, South and West Asia
*Lupinus* spp.	lupins	Australasia, Europe, Middle East, Russia
*Macrotyloma uniflorum*	horse gram	Tropical South Asia and South-East Asia
*Phaseolus lunatus*	lima bean	Temperate USA and other arid areas
*Phaseolus vulgaris*	common bean, dry beans, French bean, navy bean	East Africa, North and South America, West and South-East Europe
*Pisum sativum*	pea, field pea	Most regions of the world. Used as a green vegetable or dry grain
*Vicia faba*	faba bean, broad bean	East and West Asia, Europe, Africa. Used as a green vegetable or dry grain
*Vicia sativa*	vetch	Predominantly Africa and Europe, but also grown in the other regions
*Vigna mungo*	black gram, urad	South and South-East Asia
*Vigna radiata*	mungbean, green gram	South and South-East Asia
*Vigna unguiculata*	cowpea	Semi-arid Africa, especially Nigeria
*Vigna subterranea*	Bambara groundnut	Production restricted to Africa

*Sources*: Sprent and Sprent (1990); FAOSTAT (2021).

It is likely that nearly all these grain legumes were grown in cropping sequences that include wheat, rice or maize. Table 5 provides examples of specific legume-cereal sequences and rotations where quantitative estimates of land use areas are available. While many different combinations of crops are presented in Table 5, the large areas of soybean-maize and soybean-wheat rotations in North and South America are of most global significance (Franzluebbers et al., 2011, Rótolo et al., 2011; Table 5).

**Table 5:** Examples of major legume-cereal rotations and their estimated land area expressed in millions of hectares (Mha) for different geographic regions and countries.

Region/Country	Rotation	Area (Mha)	Reference	Comments
North America				
USA	soybean-maize	42.24^a^	www.yieldgap.org/united-states	
	soybean-rice	0.40	Carroll et al. (2020)	
Canada	pulses-wheat or canola	2.16	Statistics Canada (2011)	
South America	common bean/maize	4.2	Lithourgidis et al. (2011)	Intercropped with maize
Brazil	soybean-maize soybean-wheat	13.42 2.37	IBGE (2021); Embrapa personal communication 2021	> 90% of 2nd harvest maize follows soybean
Argentina	soybean-wheat	4.65	Calviño and Monzon (2009)	Also relay wheat-soybean-soybean double cropping
South Asia	legume^b^-rice-wheat	4.13	CGIAR (2018)	Intercropped with wheat or relay crop
Bangladesh	legume^c^-rice	0.78	Nasim et al. (2017)	
	green or black gram-wheat^d^	0.04		
	legume^e^-maize	0.02		
India	green gram-rice-wheat chickpea-rice-wheat	0.81 0.30	Atlas of cropping systems in India (2001)	
	groundnut-rice grasspea-rice black gram-rice chickpea-rice horse gram-rice pulses^f^-rice	1.03 0.95 0.60 0.30 0.25 0.12		
	chickpea-maize green gram-maize	0.40 0.16		
	soybean-wheat groundnut-wheat pulses^f^-wheat	2.23 0.16 0.10		
South-East Asia				
Philippines	green gram - cereals	0.04	Sanchez (2021)	
East Asia				
China	soybean-maize	1.1	Hu and Zimmer (2013)	
	soybean-rice	0.16	Frolking et al. (2002)	
	soybean-wheat	1.6		
	groundnut-wheat	0.9		
Africa	legume^g^/maize or other cereals	8.56	CGIAR (2018)	Intercopped with cereals
Sub-Saharan Africa	soybean-maize	1.5	Acevedo-Siaca and Goldsmith (2020)	
Europe				
Lithuania	faba bean-wheat-wheat canola-wheat-pea-wheat pea-wheat-oats	0.11	LegValue EU-project, personal communication 2021	
Oceania				
Australia	pulses^h^-wheat or canola-wheat-barley	2.26	FAOSTAT (2021): 2016–17 non-drought years average	Wheat grown on 60%, grain legumes on 10–15% of rainfed arable cropping land.
	1–4 years legume-based pasture-wheat or canola-wheat-barley	50.0	Angus and Peoples (2012); Angus and Grace (2017)	Pasture phases tend to be longer in eastern cereal belt than the west.

a Data for the USA corn-belt States (Illinois, Iowa, Indiana, Michigan, Minnesota, Nebraska, North Dakota, Ohio, South Dakota, Wisconsin).

b Legumes include are chickpea, lentil, pigeon pea, soybean and groundnut.

c Legumes include grasspea, lentil, cowpea, groundnut, soybean, green and black gram, pea and chickpea in rotation with either single rice crop (legume with single rice crop may also have another non-leguminous or non-cereal crop) or double rice crop.

d Green or black gram in rotation with either single wheat or with rice and non-rice crops.

e Legumes include green and black gram, lentil, *Sesbania* and groundnut in rotation with either single maize or rice and non-rice crops.

f Pulse species not identified.

g Legumes include cowpea, groundnut, soybean, pigeonpea, chickpea and lentil.

h Pules include chickpea (1.08 Mha), lupin (0.57 Mha), lentil (0.35 Mha), and pea (0.26 Mha).

While grain legumes are normally grown as sole crops in a sequence with cereals and non-legume oilseeds in arable agricultural soils, there are also systems where legumes and cereals are grown simultaneously together on the same land within the same growing season as intercrops (legumes and cereals sown in separate rows) or mixed/multiple crops (legumes and cereals interplanted; Lithourgidis et al., 2011; Homulle et al., 2021). Such polyculture farming systems are a common traditional practice by smallholder farmers in the rainfed cropping areas of Africa (Giller, 2001, Lithourgidis et al., 2011, Vanlauwe et al., 2019), Asia (Rekasem et al., 1988; Hobbs and Osmanzai, 2011; Raza et al., 2019) and South America (Rótolo et al., 2011, Lithourgidis et al., 2011). Maize is generally the most widely grown cereal component with a range of warm-season grain legumes; wheat and cool-season legume crop mixes are also grown in South Asia (Table 5; Hobbs and Osmanzai, 2011; Lithourgidis et al., 2011; Homulle et al., 2021). It has been suggested that these traditional multiple cropping systems might provide 15–20% of global food production (Lithourgidis et al., 2011). However, they are also a feature of some temperate organic farms (especially in Europe; Bedoussac et al., 2015; Verret et al., 2020), and are being evaluated in more conventional farming systems elsewhere in the world (Fletcher et al., 2016, Homulle et al., 2021).

#### 3.2.2 Forage legumes

Another situation where legumes play a major role in supplying N to support cereal production is in the rainfed mixed crop-livestock farming systems of Australia, which consist of alternating phases of wheat (and other grain crops) and grazed pasture leys based on self-regenerating annual clovers and medics, or alfalfa (lucerne; *Medicago sativa*: Kirkegaard et al., 2011; Angus and Peoples, 2012; Angus and Grace, 2017). While the proportion of farmland dedicated to pastures in the Australian wheat-sheep zone has declined since the 1990’s because of increasing intensification of cropping (Kirkegaard et al., 2011, Angus and Peoples, 2012), the total area of pasture grown in rotation with grain crops still represents 45–50 Mha (Table 5). Pastures are also grown on arable land in some areas of North and South America, Europe and East Asia, for the most part cereal cropping and livestock production generally occur in different parts of the landscape (Franzluebbers et al., 2011, Rótolo et al., 2011, Wolfe, 2011). Even where cropping and pasture occur on the same farm, cereal production is less closely integrated with livestock than in Australia, and each operation are often localized in different specialized areas of the farm (Rótolo et al., 2011, Wolfe, 2011).

#### 3.2.3 Green manure and cover-crops

Before the wide-spread adoption of N fertilizers in the 1960s, 25–50% of farmed land was typically committed for sowing either legume-based pastures or crops that would be grown as sources of green manure for the purpose of soil N fertility regeneration (Smil, 2001, Crews and Peoples, 2004). The legume green manures would be terminated prior to sowing the next cash crop by a combination of mowing, rolling or tillage, and the residues would either be left as a mulch or incorporated with additional cultivation. Leaving the green manure on the soil surface as a mulch had the added advantage of smothering any weeds that otherwise might regrow, as well as preventing water evaporation, thereby ensuring good soil moisture for the cash crop. This practice has continued as an essential element of organic farming (Baddeley et al., 2017): globally, all 3.3 Mha under organic cereal production (Schott and Sanders, 2017) are likely to have benefited from N supplied by green manures.

Traditionally, lowland rice farmers grew aquatic plants such as the water fern *Azolla* and semi-aquatic legumes *Sesbania*, *Aeschynomene* or *Astragalus* as N_2_-fixing green manure plants, and incorporated them by wet soil tillage (puddling) before transplanting the rice seedlings. More recent research and extension efforts have encouraged farmers to seed rice and *Sesbaia* together and then to kill the *Sesbania* after 25–30 days using the broadleaf herbicide 2,4-Dichlorophenoxyacetic acid (2, 4-D) so that the *Sesbaina* surface mulch decomposed rapidly, supplying N to the developing rice seedlings (Singh et al., 2009). Normally one or two crops of rice are grown annually in parts of Asia, and *Azolla, Sesbania* or *Aeschynomene* would be raised during the 45–60-day fallow period between rice crops as a source of N for the rice. The potential of *Azolla* and *Sesbania* to produce biomass dry matter yields of 6–8 t ha^-1^ is roughly equivalent to an application of 100–200 kg N ha^-1^ as urea (Ladha et al., 1992). However, the use of aquatic green manure (*Azolla* and legumes) by farmers in many countries of Asia has gradually declined since the 1980 s in favor of crop intensification (Roger and Watanabe, 1986). Currently there is no clear advantage for rice farmers to choose *Azolla* or *Sesbania* as a source of N for rice over N fertilizer because the additional costs of labor, land opportunity, irrigation, seed/inoculum, phosphate and pesticides makes their use uneconomical.

Until recently, strategies of using sacrificial green manure crops also made little economic sense in most conventional wheat and maize cropping systems. The additional N benefits gained rarely justified the potential income forgone in a green manuring year. However, the evolution of weeds that have resistance to multiple herbicide molecules has forced conventional farmers in different parts of the world to re-evaluate growing low-input legumes (e.g., vetch or field pea) that can be green manured or killed with knock-down herbicide as “brown manure”. This is because the development of resistance has meant that farmers may encounter additional time and management challenges associated with assuring knock-down before herbicide-resistent weeds can set viable seed as an additional strategy to help control intractable weeds (Llewellyn et al., 2016). There is little quantitative information about the prevalence of such practices globally, but farmer surveys suggest that green/brown manuring is periodically utilized nationally as a weed control strategy by around one-quarter of Australian grain-growers on 15% of their cropping land, an area equivalent to ~0.75 Mha (Llewellyn et al., 2016).

Cover-cropping is another variation of green manuring that is increasingly being used by both organic and conventional farmers. This is particularly the case in the temperate climatic zones of Europe and North America, where the summer period is used primarily for crop production and winter is when the highest percolation of water through the soil occurs due to low evaporation and high precipitation (Baddeley et al., 2017, Kaye and Quemada, 2017). After harvest of the main crop in these agroecological systems the temperature and light conditions are still sufficient to support some plant growth, although not enough to produce a commercial cash crop. Rather than leaving cropping land bare, cover crops of legumes (either alone or mixed with non-legumes) are now increasingly sown to lower the soil inorganic N content to reduce the risk of leaching and denitrification events during the fallow period (Mueller and Thorup-Kristensen, 2001, Thorup-Kristensen et al., 2003, Plaza-Bonilla et al., 2017). After the cover crop is incorporated prior to sowing the next spring-summer crop, it effectively becomes a source of green manure and provides N benefits for subsequent crops (Baddeley et al., 2017).

There are analogous uses of short-duration legume cover crops in the tropics. For example, in lowland rice production, cover crops (also called catch crops) may be grown between successive rice crops to capture inorganic N that otherwise would be lost with land flooding (George et al., 1994, Ladha et al., 1996, Shrestha and Ladha, 1998), and in southern Brazil the use of legume cover crops is increasingly common in no-tillage systems (Mielniczuk et al., 2003). Elsewhere, smallholder farmers sow legume cover crops in rain-fed systems, either prior to the maize harvest (relay cropping) or after maize maturity to produce a dense mat of organic material to suppress weeds, and to stabilize the soil and protect it from erosion on sloping lands (Giller and Cadisch, 1995, Giller, 2001).

## 4 Estimates of BNF

Different methodologies have been deployed in the study of BNF by free-living and plant-based N_2_-fixing systems (Table 6). These methods either aim to:

(a) measure the rate of nitrogenase activity using either ^15^N_2_ feeding (Boddey, 1987, Chalk et al., 2017) (this technology can also be used to monitor the transfer of fixed N from free-living diazotrophs to cereals; Giller et al., 1984), the acetylene reduction assay (Hardy et al., 1968, Boddey, 1987, Witty et al., 1979) or hydrogen evolution (Hunt and Layzell, 1993, Unkovich et al., 2008),
(b) provide a short-term assessment of the percentage of plant N derived from atmospheric N_2_ (%Ndfa) through analysis of xylem sap collected to provide ureides allantoin and allantoic acid (McClure et al., 1980, Herridge et al., 1990, Herridge and Peoples, 2002),
(c) allow a time-intregrated estimate of %Ndfa over a period of growth using ^15^N isotope-dilution based on either ^15^N-enrichment technologies (Witty, 1983, Pareek et al., 1990, Chalk and Ladha, 1999, Chalk, 2016) or ^15^N natural abundance analysis (Shearer and Kohl, 1986, Malarvizhi and Ladha, 1999, Alves et al., 2014, Chalk and Craswell, 2018), or
(d) determine inputs of fixed N_2_ derived either by comparing differences in N-uptake between N_2_-fixing and non-N_2_-fixing systems undertaken on either an annual basis (N-difference: Unkovich et al., 2008), or from calculations based on measuring quantitative changes in total soil N combined with data documenting multiple sources of N inputs, removal and losses collected over many years (N balance: Jenkinson, 1977; Powlson et al., 1986; Chalk, 1998b; Ladha et al., 2000; Pampolina et al., 2008; Ladha et al., 2016).

**Table 6:** Different methods used to study BNF by N_2_-fixing systems under controlled-conditions or in the field^a^.

Method	Principle	Advantages	Disadvantages	Extent of use
^15^N_2_ feeding	Intact or detached plants roots and/or nodules, or soil placed in a chamber with an atmosphere enriched in ^15^N_2_. The amount of ^15^N accumulated at the end of a period of incubation provides a direct measure of the rate of N_2_ fixation	Direct measure of N_2_ fixation The only technique apart from growing plants in N-free medium under controlled-conditions to unequivocally prove active N_2_ fixation	High cost of ^15^N_2_ gas Requires high-level technical skills Measurements reflect nitrogenase activity only for the duration of assay Can’t distinguish between N_2_ fixed by free-living diazotrophs in the soil, or on the external surface of plants, from that occurring within the plant Can be difficulties keeping incubation systems completely sealed while maintaining suitable environmental conditions (e.g. temperature and O_2_ levels) inside the chamber Difficult to use under field conditions Errors can arise due to the contamination of^15^N_2_ gas with traces of other ^15^N-compounds that can be assimilated by microbes or plants Not suitable for long-term determinations of BNF	Limited use because of the logistical difficulties
Acetylene reduction	The enzyme nitrogenase, which reduces N_2_ to NH_3_ is also capable of reducing acetylene (C_2_H_2_) to ethylene (C_2_H_4_). If roots, nodules, or soil are placed in an airtight vessel or contained within a cuvette connected to a flowing gas-stream, and then exposed to a C_2_H_2_ enriched atmosphere, the accumulation of C_2_H_4_ over a period of assay is used to provide an indirect assessment of the rate of BNF	Sensitive diagnostic tool for detecting nitrogenase activity Simple, rapid, and relatively inexpensive, and many measurements can be undertaken daily	Requires a gas chromatograph to quantify the concentration of C_2_H_4_ in gas samples C_2_H_2_ is explosive and poses a possible hazard Establishment of flow-through gas exchange to monitor intact systems is extremely difficult Difficult to use under field conditions Commonly applied to detached roots (or nodules) rather than whole root systems, so total BNF will be underestimated Errors due to changes in gas exchange induced by disturbance of the N_2_-fixing system for assay Uncertainties about the appropriate conversion ratio to apply to calculate the amount of N fixed from C_2_H_2_ reduction data. Ideally should be calibrated with^15^N_2_ Provides only a short-term estimate of BNF. Multiple, repeated measurements are required to monitor BNF over a growing season The underlying assumptions that substituting C_2_H_2_ for N_2_ does not affect nitrogenase activity, and that measures obtained under assay conditions are related to BNF rates in situ do not hold for legume nodules	Considered unreliable for nodulated legumes, but still used to assess BNF by non-symbiotic systems and free-living diazotrophs
Hydrogen evolution	Hydrogen (H_2_) gas is an obligate by-product of BNF in legume nodules. An indirect measure of nitrogenase activity can be obtained by placing a nodulated legume root system in a cuvetter and monitoring the increase in H_2_ concentration in a gas-stream	Sensitive diagnostic tool for detecting nitrogenase activity Measurements of H_2_ evolution in air do not inhibit nitrogenase activity so repeated assays can be performed on the same plant material Simple, rapid, and inexpensive	Requires a gas chromatograph or H_2_-electrode to quantify the concentration of H_2_ in gas samples H_2_ evolution in air measures only represents a portion of total electron flux through nitrogenase Some rhizobial strains form symbioses that have an active hydrogenase uptake enzyme that recycles H_2_ within the nodule, so no H_2_ will be detected despite BNF occurring To measure total nitrogenase activity it is necessary to incubate nodulated roots in the absence of N_2_ (e.g. argon:oxygen) rather than in air Difficulties in establishing flow-through gas exchange systems to monitor roots of intact plants Difficult to use under field conditions Commonly applied to detached nodulated roots (or nodules) rather than whole root systems, so total BNF will be underestimated	Predominantly applied to nodulated legumes in the laboratory or in controlled-environment experiments. Potential use in non-legume systems largely unexplored
Sap-ureide	The forms of N transported in the xylem stream from N_2_-fixing nodules (ureides) differs from soil N assimilated by roots (amino acids and NO_3_) in some legume species. Consequently, analysis of the N-solute composition in xylem sap (or plant tissue) can be used to assess the percentage of plant N derived from atmospheric N_2_ (%Ndfa)	Rapid and involves simple colorimetric assay of either xylem sap or tissue extract in a test tube Not technically difficult No special experimental design required Suitable for well-watered experimental and farmers’ legume crops	Restricted to ureide-exporting legume crops including soybean, pigeon pea, *Vigna* and *Phaseolus* species Requires calibration with another method such as^15^N-isotope dilution Calibration relationships are crop specific and may change with growth stage Provides only a short-term estimate of %Ndfa, so multiple, repeated measurements usually required over the duration of the growing season	Can only be applied to certain sub-tropical legume species Not suitable for temperate legumes
^15^N isotope-dilution	If the^15^N concentration in atmospheric N_2_ differs significantly from that of plant-available soil N, %Ndfa can be calculated from a comparison of^15^N composition of a N_2-_fixing plant-based system with a non-N_2_-fixing reference plant(s) Assumes reference plant provides a surrogate measure of the^15^N signature of the same plant-available soil N pool used by N_2_-fixing plant	Provides a “time-integrated” estimate of %Ndfa over the period of growth Amounts of N_2_ fixed can be estimated from a single analysis of plant material for^15^N and %N contents If^15^N natural abundance of soil N is sufficiently high and uniform, can be applied to both experimental and farmers’ crops	Requires non-N_2_ fixing reference plants ideally with similar rooting depths and patterns of N uptake to that of N_2_-fixing plant Prone to errors if^15^N composition of plant-available soil N changes markedly with soil depth or with time during the growing season High cost of^15^N-enriched materials if they are used to expand the difference between the^15^N composition of soil mineral N and atmospheric N_2_ With^15^N natural abundance there is a need to account for isotopic-fractionation that results in a slight depletion of^15^N in shoots of legumes fully dependent upon BNF for growth when calculating %Ndfa. ^15^N natural abundance cannot be used to estimat%Ndfa of nodulated roots as isotopic-fractionation results in^15^N accumulation in nodules	Widely used in both non-symbiotic and symbiotic N_2_-fixing systems
N-difference	Nitrogen difference compares legume accumulation of N with that of a neighboring non N_2_-fixing crop or plant over a single growing season. The difference in N between the two is assumed to be due to N_2_ fixation	Simple, low-cost method that can be applied when facilities for total N analyses are available	Calculations are highly dependent on the accuracy of sampling for the determination of plant biomass and sub-sampling for %N analysis Errors can arise if the amount of soil mineral N accumulated by the non N_2_-fixing control plant differs markedly from that of the N_2_ -fixing plant Most reliable in low soil N fertility soils when BNF is high Not suitable for measurement of non-symbiotic BNF because of difficulties in quantifying low levels of N_2_ fixation	Widely used in legume systems
N-accretion or N balance	All possible external inputs (fertilizer, manures, wet and dry deposition, N in irrigation water, ammonia absorportion by leaves) and outputs of N (N removed in plant or animal products, leaching, run-off and erosion, volatilization, denitrification) need to be accounted for, and incremental changes in soil N quantified in the system under study. If a net positive total N balance is calculated to occur between two points in time, then the N gain this is attributed to inputs of fixed N_2_	Can potentially be applied to experiments and farmers’ fields	N outputs through various loss processes are difficult and complex to quantify and often rely on assumptions rather than actual measurements Quantification of some N inputs (e.g. atmospheric deposition of N) can be challenging Quantification of changes in soil N pool is subject to large errors, substantial inputs from BNF are necessary to reliably quantify any increase in soil N As the method relies on many independent and unrelated measurements, each made with differing degrees of accuracy, the confidence in the final estimate of BNF can be low	Use is limited to long-term studies for both non-symbiotic and symbitoic N_2_-fixing systems

a Adapted and updated from information provided by Unkvoich et al. (2008); Peoples et al. (2009b); Chalk et al. (2017); Soper et al. (2021).

All these different approaches are technically challenging with their own unique strengths and limitations, and each are subject to specific potential sources of error when being applied to either symbiotic (Minchin et al., 1983, Giller, 2001, Unkovich et al., 2008, Peoples et al., 2009b, Chalk et al., 2016), or non-symbiotic systems (Boddey, 1987, James, 2000, Santi et al., 2013, Bellenger et al., 2014, Unkovich et al., 2020, Soper et al., 2021; Table 6). Consequently, putative evidence of BNF, even by nodulating legumes, should ideally not rely solely upon the application of a single measurement technique. Also, BNF studies should not be undertaken without the inclusion of treatments and the collection of additional data to (a) provide supporting evidence to confirm a greater accumulation of biomass N by the presumed N_2_-fixing system compared to a non-fixing control, and (b) to rule-out alternative explanations other than BNF for the observed results (Giller and Merckx, 2003, Unkovich and Baldock, 2008, Unkovich et al., 2020, Soper et al., 2021). As highlighted in a commentary by Unkovich et al. (2020) concerning the inappropriate application of the ^15^N natural abundance methodology, this is particularly important in studies where amounts of fixed N_2_ reported for a non-legume that was not previously known for its N_2_-fixing capabilities exceed the rates commonly achieved by legumes. However, it should be acknowledged that researchers do not always publish inconclusive or negative results, so the frequency of low or negligible levels of BNF could well be under-reported. Nevertheless, such information is arguably just as valuable in advancing scientific knowledge and understanding as high BNF values measured in “hotspots” or during temporal “hot moments” of BNF activity (Soper et al., 2021).

Various BNF data generated by most of the techniques described in Table 6 is utilized in the following sections when describing the contributions from different sources of N_2_ fixation in cereal-based cropping systems. The main exception will be acetylene reduction, which has been discredited for use with nodulated legumes (Minchin et al., 1983, Giller, 2001, Unkovich et al., 2008, Peoples et al., 2009b). Despite similar concerns also expressed about the reliability of the method to provide quantitative estimates of BNF for free-living and non-symbiotic N_2_-fixing systems (e.g., Boddey, 1987; James, 2000; Giller and Merckx, 2003; Unkovich and Baldock, 2008), the method is still used, because of the paucity of viable alternatives (Soper et al., 2021). Consequently, some of the following narrative concerning non-symbiotic BNF by necessity, includes information derived from acetylene reduction due to the lack of alternative data from other methodologies.

### 4.1 Global

Although much attention is given to synthetic fertilizers, BNF is the largest single global input of reactive nitrogen (Fowler et al., 2015). Broadly, there are three ecosystems which receive substantial inputs of biologically fixed N: natural (unmanaged) terrestrial systems, aquatic/marine environments, and agricultural systems. A wide range of estimates of BNF have been reported for each of these distinct ecosystems, reflecting the large number of uncertainties and unknowns which result from extrapolating insufficiently reliable or representative measurements of BNF to a global scale (Galloway et al., 2004, Fowler et al., 2015, Davies-Barnard and Friedlingstein, 2020, Soper et al., 2021). While there are likely to be flows of N from natural terrestrial and aquatic ecosystems to agriculture and vice versa, the following discussions focus primarily on inputs of BNF in arable croplands.

Table 7 presents a comparison of global estimates of BNF in agriculture provided by grain legume-rhizobium symbioses, and/or free-living/endophyte/symbiotic associations in rice and other major cereals. The calculated estimates of BNF by grain legumes increased from 10 Tg N in the mid-1990s (Smil, 1999) to 35.5 Tg N by 2018 (Herridge et al., 2022). This growth was largely associated with increased areas of grain legume production over time. Of the 35.5 Tg N deemed to be fixed in 2018, soybean accounted for 70% of the total. The estimates of BNF for non-leguminous systems reported by Smil (1999), Herridge et al. (2008) and Battye et al. (2017) were comparable (approximately 10 Tg N), but a 50-year (1960–2010) global N budgeting assessment undertaken by Ladha et al. (2016) calculated that annual inputs of BNF from non-symbiotic sources in cereal cropping systems may have represented 14.8 Tg of fixed N in 2010 (3.3, 5.6 and 5.9 Tg N yr^-1^ each for wheat, rice and maize, respectively). The extrapolated estimates of non-symbiotic BNF calculated by the authors for 2018 by applying the relationships developed by Ladha et al. (2016) to updated production data was 15.7 Tg of fixed N (3.1, 5.7 and 6.9 Tg N yr^-1^ each for wheat, rice and maize, respectively). The change in estimates of BNF by maize and rice were due to increases in total harvested areas (by 18.2% and 3.7% for maize and rice, respectively) from 2010 to 2018, and a decrease in wheat harvested area (1.3%).

**Table 7:** Estimates of annual inputs of BNF (Tg N) by symbiotic and non-symbiotic diazotrophs in cereal-based cropping systems and the year(s) for which the values were calculated.

Crop system	Diazotroph	Smil (1999)	Herridge et al. (2008)	Ladha et al. (2016)	Battye et al. (2017)	Herridge et al. (2022)
		1994–96	2005	2010	2013	2018
Grain legumes	Rhizobium-legume symbiosis	10 (8–12)	21.5^a^		32.5^b^	35.5^c^
Rice cultivation	free-living, endophytic and/or symbiotic (*Azolla*)	5 (4–6)	5	5.6^d^	10	
Cereals other than rice Cereals including rice	free-living, endophytic	4 (2–6)	< 4	9.2^e^		

a Soybean 16.4 and other legumes 5.1 Tg N.

b Soybean 25 and other legumes 7.5 Tg N.

c Soybean 25.0 and other legumes 10.5 Tg N.

d Updated value for 2018 calculated to be: 6.9 Tg N.

e Maize 5.9 and wheat 3.3 Tg N in 2010; updated values for 2018 calculated to be: maize 5.7 and wheat 3.1 Tg N.

### 4.2 Non-symbiotic BNF in wheat, rice and maize systems

Non-symbiotic N_2_ fixation during cereal cropping takes place in soil, the plant-soil surface, and inside the plant. These can be referred to as autochthonous (indigenous) BNF systems comprising heterotrophic and phototrophic bacteria and cyanobacteria native to soil-plant-floodwater (Roger and Ladha, 1992, Giller, 2001, Reddy et al., 2002, Bageshwar et al., 2017, Van Deynze et al., 2018).

Nitrogen fixed by free-living diazotrophs largely enters the SON pool after the micro-organisms die before becoming available for crop uptake. In addition to that N contributed after microbial death, a portion of the N_2_ fixed by bacteria associated with a crop may also become available to the plant (Giller et al., 1984). The proof of principle that free-living N_2_ fixation occurs in rice, maize and wheat systems (and other cereals) has been demonstrated using various N_2_ fixation measurement methodologies, including ^15^N_2_ feeding (SI Appendix A, 3.2.1, 3.2.2, 3.2.3, 4.5.1, 4.5.2, 5.1.1, 5.1.2, 5.2.1, 5.2.2, 5.2.3, 5.2.4, 5.2.5, 3.1, 3.2, 4.1, 4.2, 4.3, 4.4, 4.5, 5.1, 5.2, Table 1, Table 2, Table 3, Table 4, respectively) and *nif* (N_2_-fixing) gene analysis (i.e., Knauth et al., 2005). Chalk (2016) undertook an extensive review of many studies reporting endophytic N_2_ fixation. These studies were mostly carried out over short durations on a small scale (e.g., growth chambers, pots with cultural media and soil) with and without inoculation of diazotrophs (SI Appendix A, 3.2.1, 3.2.2, 3.2.3, 4.5.1, 4.5.2, 5.1.1, 5.1.2, 5.2.1, 5.2.2, 5.2.3, 5.2.4, 5.2.5, 3.1, 3.2, 4.1, 4.2, 4.3, 4.4, 4.5, 5.1, 5.2, Table 1, Table 2, Table 3, Table 4) or attempts to identify apparent genetic differences in BNF associations across crop germplasm (SI Table S5). However, some data come from a mix of controlled environment and field investigations using multiple measurement techniques, such as the recent report that 29–82% of N acquired by a Mexican indigenous maize landrace grown on unfertilized N-deficient soil over five years appeared to be derived from BNF (Van Deynze et al., 2018: SI Table 3). Based on the estimates of %Ndfa and the amounts of N accumulated by crops it was calculated that inputs of BNF could have represented up to 122 kg N ha^-1^ yr^-1^, although at most locations determinations were within the range of 4–15 kg N ha^-1^ yr^-1^ (Van Deynze et al., 2018).

Most inoculation experiments include uninoculated treatments as “non-fixing” references, while in ^15^N-based studies comparing crop germplasm either the cultivar exhibiting the lowest ^15^N excess or the measured ^15^N excess of soil available N have been used as reference controls. While estimates of extraordinary high %Ndfa values have been reported (highest values for rice 59%, maize 82% and wheat 85%; SI Appendix A, 3.2.1, 3.2.2, 3.2.3, 4.5.1, 4.5.2, 5.1.1, 5.1.2, 5.2.1, 5.2.2, 5.2.3, 5.2.4, 5.2.5, 3.1, 3.2, 4.1, 4.2, 4.3, 4.4, 4.5, 5.1, 5.2, Table 1, Table 2, Table 3, Table 4), most %Ndfa determinations for inoculation or cultivar comparisons have been < 33% across all three crops (SI Appendix A, 3.2.1, 3.2.2, 3.2.3, 4.5.1, 4.5.2, 5.1.1, 5.1.2, 5.2.1, 5.2.2, 5.2.3, 5.2.4, 5.2.5, 3.1, 3.2, 4.1, 4.2, 4.3, 4.4, 4.5, 5.1, 5.2, Table 1, Table 2, Table 3, Table 4, Table 5). Although the higher reported %Ndfa values or measured N gains based on total N balance (e.g., App et al., 1986; SI Table S5) could be considered as being indicative of potential upper limits for BNF in these cereal systems, much of these data come from pot experimentation and should not be directly extrapolated to estimates of BNF on a hectare basis.

In addition to free-living heterotrophic diazotrophs, the submerged soils within which most rice is grown provide a conducive environment for phototrophic (e.g., cyanobacteria) BNF. Under favorable conditions, cyanobacteria can fix 20–40 kg N ha^-1^ crop^-1^ (Roger and Watanabe, 1986). Since the discovery of the importance of cyanobacteria in contributing to N gains under flooded conditions, many inoculation experiments have been conducted using indigenous or cultured cyanobacteria as prospective strategies to improve soil fertility and grain yield in rice. Based on extensive review of literature, Roger (1991) concluded that the effect of cyanobacterial inoculation on rice yields ranged from 0 to 3.7 t ha^-1^ (average increase of 337 kg grain ha^-1^ or 11.3% increase compared to without inoculation). However, studies undertaken in numerous rice fields in Asia showed that indigenous diazotrophic cyanobacteria were widely present and that inoculated non-indigenous strains rarely become established (Reddy and Roger, 1988).

The methods used for BNF measurements of free-living/endophytic diazotrophs integrate N_2_ fixation occurring in soil with whatever might be occurring in association with plants, and it is difficult to distinguish between the fixed N arising outside or inside a plant. Nevertheless, researchers have often attributed the integrated estimates to fixation by endophytes (see review by Chalk, 2016). While many studies have established that non-symbiotic N_2_ fixation occurs in the soil-plant habitat, most of them do not represent actual field settings, for multiple reasons. Trials were often undertaken at small scale, sometimes crops were also not grown to maturity, and experimental constraints may have resulted in sub-optimal plant growth conditions.

It is argued that the determination of the N balance of long-term field experiments represents a logical initial approach to estimate the contributions of BNF by non-symbiotic diazotrophs to the N economies of agroecosystems. Several studies in rice where N balance calculations were constructed taking all known N inputs into account, have indicated positive N balances ranging from 18 to 51 kg N ha^–1^ crop^–1^. There have been fewer similar undertakings for other cereals, but net N balances have been reported from 13 to 35 kg N ha^–1^ crop^–1^ for wheat and 13–26 kg N ha^–1^ crop^–1^ for maize (Table 8). However, it is important to note that the derived N balance data were the sum of measured inputs and outputs, and often no N losses were measured or included in the calculation. Consequently, actual contributions of BNF could be underestimated. There are some exceptions. For example, BNF estimates derived from the 50-year cereal N-budgeting exercise reported by Ladha et al. (2016) did consider N losses in the calculations.

**Table 8:** Field estimates of the inputs (kg N ha^-1^ per crop) of non-symbiotic/free-living BNF in rice, wheat, and maize systems.

Location	Annual crop rotation	Crop	No. of crops	Year	Method	Input of fixed N	Remarks	Reference
Jiangdu, China	rice-rice	rice	1	N/A^a^	^15^N_2_ feeding	4–19^b^	direct method (90 d^15^N_2_ incubation)	Zhang et al. (2021)
Jiangdu, China	rice-rice	rice	1	N/A^a^	^15^N_2_ feeding	11	direct method (28 d^15^N_2_ incubation)	Wang et al. (2020)
Jiangdu, China	rice-wheat	rice	1	N/A^a^	^15^N_2_ feeding	22–51	direct method (74 d^15^N_2_ incubation)	Ma et al., 2019a, Ma et al., 2019b
Jiangdu, China	rice-wheat	rice		2010	^15^N_2_ feeding	45	direct method (70 d^15^N_2_ incubation)	Bei et al. (2013)
Global average	single to multiple	rice	50	1960–2010	N balance	22	excluding deposition and seed N	Ladha et al. (2016)
Bocol, Philippines	rice-rice	rice	30	1968–83	N balance	35	excluding deposition	Pampolina et al. (2008)
Los Banos, Philippines	rice-rice	rice	30	1964–79	N balance	18	excluding deposition	Pampolina et al. (2008)
Maligaya, Philippines	rice-rice	rice	30	1968–83	N balance	44	excluding deposition	Pampolina et al. (2008)
Los Banos, Philippines	rice-rice-rice	rice	45	1963–83	N balance	27	excluding deposition	Pampolina et al. (2008)
Los Banos, Philippines	rice-rice	rice	27	1985–98	N balance	46	excluding deposition	Ladha et al. (2000)
Pakistan		rice	1	N/A^a^	^15^N dilution	46	including deposition	Malik et al. (1997)
Global average	Rice	rice	N/A	N/A^a^	N balance	30	excluding deposition	Roger and Ladha (1992)
Japan	Rice	rice	1	N/A^a^	N balance	40–45	including deposition	Marumoto (1986)
Los Banos, Philippines	rice-rice	rice	24	1966–78	N balance	51	excluding deposition	App et al. (1984)
Maligaya, Philippines	rice-rice	rice	17	1968–1976	N balance	39	excluding deposition	App et al. (1984)
IARI, India		wheat	1	N/A^a^	N balance	40	excluding deposition and seed N	Bageshwar et al. (2017)
Global average	single to multiple crop	wheat	50	1960–2010	N balance	13	excluding deposition and seed N	Ladha et al. (2016)
Avon, Australia	Wheat	wheat	17	1979–96	N balance	20	excluding deposition and seed N	Gupta et al. (2006)
Rothamsted, U.K.	Wheat	wheat	4	1979–83	N balance	25	excluding deposition and seed N	Powlson et al. (1986)
Rothamsted, U.K.	Wheat	wheat	115	1852–1967	N balance	25–35	excluding deposition and seed N	Jenkinson (1977)
Rio de Janerio, Brazil	Maize	maize	3	N/A^a^	^15^N dilution	26		Alves et al. (2014)
Global average	single to multiple crop	maize	50	1960–2010	N balance	13	excluding deposition and seed N	Ladha et al. (2016)

a Not available.

b 19.25 kg N ha^-1^ reported under no-N and 2.67–3.61 kg N ha^-1^ with N-fertilizer (at the rates of 125–250 kg N ha^-1^).

Nevertheless, it is important to acknowledge that measurements of non-symbiotic N_2_ fixation derived from soil/plant N balance calculations based on many independent and unrelated measurements, each made with a differing degree of accuracy, will inevitably entail errors (Giller and Merckx, 2003, Unkovich and Baldock, 2008, Chalk, 2020). Additionally, it does not indicate whether fixed N is translocated from the site of fixation (roots and stem) to the above-ground biomass and grain within a cropping season. Although ^15^N_2_ feeding is the only direct means of conclusively quantifying fixation, the method has rarely been utilized to monitor BNF over an entire growing season up to crop maturity because of its short-term nature, the overwhelming technical challenges, and cost (Table 6). However, ^15^N_2_-labeling ﬁeld-based growth chamber studies have been undertaken in China (Bei et al., 2013, Ma et al., 2019a, Ma et al., 2019b, Wang et al., 2020, Zhang et al., 2021) which included measurements of BNF by rice plants grown close to maturity and those estimates of non-symbiotic N_2_ fixation ranged from 19 to 51 kg of N ha^–1^ crop^–1^. One of these studies (Ma et al., 2019a) calculated 23 and 39 kg of fixed N ha^−1^ for the whole plant-soil systems with inbred japonica (W23) and hybrid indica (IIY) rice cultivars, respectively, but only 1–2.5% of this fixed N was detected in rice plants or weeds. This was consistent with earlier conclusions that much of the non-symbiotic fixed N_2_ enters via SON rather than directly supporting the nutrition of the current crop. Interestingly, high throughput sequencing of *nifH* genes extracted from surface soil showed that the presence of rice affected the community composition of diazotrophs (Wang et al., 2020). The relative abundance of the Nostocales and Stigonematales was significantly higher in rice-planted soil than in non-planted soil. Further studies are needed to decipher what influence plant type or crop variety may have on non-symbiotic N_2_ fixation and to elucidate possible mechanism(s) of interaction. It is important to note that except for the study by Zhang et al. (2021) all other investigations were carried out in soil without application of synthetic N, which is atypical of most rice production systems. Zhang et al. (2021) reported an 81–86% reduction in rates of N_2_ fixation when fertilizer N was applied at rates of 125–250 kg N ha^-1^, yet *nifH* copy number increased. Similar ^15^N_2_ studies are also needed for wheat and maize under common farmer N fertilization practices.

### 4.3 Symbiotic BNF (aquatic green manures) in rice cultivation

Because of their aquatic environment, rice lowlands provide favorable conditions for the water fern *Azolla*, which harbors symbiotic N_2_-fixing cyanobacteria *Anabaena azollae,* and aquatic legumes such as *Sesbania* and *Aeschynomene* spp. that form symbioses with heterotrophic and phototrophic rhizobia (Ladha et al., 1992). These allochthonous (exogenous) BNF systems comprising *Azolla* and legumes are not ubiquitous in agriculture and hence need to be introduced to rice fields to provide additional N to the crop (Reddy et al., 2002).

*Azolla* is generally inoculated and grown as a cover-crop with or without rice for incorporation into the soil as a top-dressing in rice cultivation. Under optimum conditions, up to 99% of *Azolla* N can be derived from the atmosphere (Yoneyama et al., 1997) and substantial amounts of BNF can be fixed, as much as 70% of which becomes available to the rice crop upon incorporation (Roger and Ladha, 1992). A single standing crop of *Azolla* in a field can accumulate from 20 to 146 kg N ha^-1^ (average 70 kg ha^-1^), and the N_2_-fixing rate can range from 0.4 to 3.6 kg N ha^-1^ d^-1^ (average 2 kg N ha^-1^ d^-1^) in a growing cycle of approximately 40 days (Becker et al., 1995). In addition to the huge BNF contribution of *Azolla*, its soil cover also reduces NH_3_ volatilization losses (Vlek et al., 1995).

Up to 458 kg N ha^-1^ has been reported to be fixed by green manured aquatic legumes, but more typically BNF ranged between 100 and 180 kg N ha^-1^ (Becker et al., 1990). Pareek et al. (1990) observed that%Ndfa in well-nodulated *S. rostrata* and *S. cannabina* increased with plant age from 50% to 75% at 25 days after seeding, 70–95% at 45–55 days, and close to 100% by 65 days. Although %Ndfa tended to be similar in both *Sesbania* species, the amount of N_2_ fixed was greater for *S. rostrata* because of higher N accumulation in biomass*. A. afraspera* is less photoperiod-sensitive than *S. rostrata*, and therefore superior in N accumulation and BNF during periods of the year when days are shorter (Becker et al., 1990).

### 4.4 Symbiotic BNF by legumes

A diverse range of cool and warm season legume grains, forage, green manure and cover crops is grown in cereal-based farming systems. Regional estimates of N_2_ fixation (%Ndfa and total amounts of fixed shoot N) along with total hectarage and production by grain legumes of major importance are provided for 2019 in Table 9. The general trends in BNF by the different legume species are consistent with previous observations (Walley et al., 2007; Salvagiotti et al., 2008; Peoples et al., 2009a). Regional average %Ndfa values ranged from as low as 36–41% (common bean, green and black bean) to 77–87% (faba bean, pigeonpea), with the amounts of fixed shoot N varying from 25 to 29 kg (cowpea, common bean) to 221 kg of N ha^-1^ crop^-1^ (faba bean) across different geographic regions (Table 9). The observed variability among species and regions in the amounts of N_2_ fixed is to be expected because of wide differences in breeding effort and cultivars grown (genetics, G_L_), rhizobial strains used in inoculants or present in the soil (genetics, G_R_), environment (E), and agronomic practices (Management, M), and the interactions between G_L_ × G_R_ × E × M (Giller and Cadisch, 1995, Herridge and Danso, 1995, Peoples et al., 1995b, Vanlauwe et al., 2019). Nevertheless, when considered on a species basis, average %Ndfa appeared to be relatively consistent across geographic regions for most crop species except soybean (44% in Europe to 78% in Brazil; Peoples et al., 2021; Table 9).

**Table 9:** The relative contributions by various geographic regions to total global grain harvested for widely grown grain legumes in 2019, the mean regional estimates of the percentage of legume N derived from atmospheric N_2_ (%Ndfa) and amounts of shoot N fixed^a^.

Legume species^b^		Geographic region contribution	Mean Ndfa	Amount shoot N fixed
	(% global grain production)^b^		(%)	(kg N ha^-1^ crop^-1^)
Soybean 120.5 Mha, 333.7 Mt	South America (55%)^c^ North America (31%)^d^ East Asia (5%) South Asia (4%) Europe (3%) South-East Asia (1%) Africa (1%)		72 62 55 56 44 67 56	177 157 76 114 128 119 78
Groundnut 29.6 Mha, 48.8 Mt	East Asia (36%)^e^ Africa (34%) South Asia (14%) Americas (10%) South-East Asia (6%)		61 57 66 68 62	101 62 91 103 121
Green & black gram 18.2 Mha, 14.5 Mt	South-East Asia (41%) South Asia (37%) East Asia (21%)^e^ Oceania (1%)		59 61 54 41	65 45 50 41
Common bean 14.9 Mha, 14.4 Mt	Africa (49%) South America (30%) North America (19%) Europe (2%)		36 39 37 39	29 35 36 60
Cowpea 14.5 M ha, 8.9 Mt	Africa (97%) Asia (2%) Americas (1%)		62 50 52	47 25 39
Chickpea 13.7 Mha, 14.3 Mt	South Asia (73%) West Asia (10%) North America (6%) Africa (5%)^e^ Europe (4%) Oceania (2%)		78 58 55 62 68 58	51 41 64 56 54 69
Field pea 7.3 Mha, 14.2 Mt	Europe (37%) North America (37%) Asia (18%) Africa (4%)^e^ South America (2%) Oceania (2%)		68 56 61 64 73 62	118 81 120 95 197 80
Pigeon pea 5.6 Mha, 4.4 Mt	South Asia (75%) Africa (15%) South-East Asia (8%)^e^ Americas (2%)^e^		67 87 77 77	123 60 92 92
Lentil 4.8 Mha, 5.7 Mt	North America (42%) South Asia (30%) Oceania (9%) West Asia (8%) South America (3%)^e^ Africa (3%) East Asia (3%)^e^ Europe (2%)		63 67 66 66 63 54 63 60	70 50 91 86 83 130 83 68
Faba bean 2.6 Mha, 5.4 Mt	East Asia (32%) Europe (29%) Africa (27%)^e^ Oceania (6%) North America (4%) West & South Asia (2%)		62 77 71 73 74 78	221 137 148 130 103 151
Lupin 0.9 Mha, 1.0 Mt	Oceania (47%) Europe (39%) Africa (8%)^e^ South America (6%)		67 73 69 68	121 151 130 118
Vetch 0.4 Mha, 0.8 Mt	Africa (43%)^e^ Europe (31%) Americas (13%) Asia (12%) Oceania (1%)		69 64 75 63 58	81 59 45 136 82
Bambara groundnut 0.4 Mha, 0.2 Mt	Africa (100%)		56	48

a Calculated from 5374 experimental and on-farm estimates of BNF collated from the 328 publications and unpublished sources cited by Peoples et al. (2021).

b Production data reported by FAOSTAT (2021) for 2019 rounded to closest whole percentage number.

c Weighted mean estimate of %Ndfa for South America was derived from BNF data reported for Brazil (34% of total global production, average %Ndfa = 78) and Argentina (17% of production, average %Ndfa = 63). For the sake of the calculation, it was assumed the remaining 4% of global production from South American countries where no BNF field data were available had the same %Ndfa as Argentina. Average amounts of shoot N fixed in Brazil and Argentina were calculated to be very similar (178 and 176 kg N ha^-1^; respectively).

d Calculated for USA experimental soybean trials undertaken between 2000 and 2017.

e Where no regional data were available the presented values represent the species global average.

The lowest inputs of BNF were recorded for common bean and *Vigna* species, green and black gram, cowpea and Bambara groundnut, which fixed on average between 30 and 50 kg of N ha^-1^ (Table 9). In the case of common bean, this was due to a low inherent capacity for BNF (global average %Ndfa of 37%), but for the *Vigna* species the low amounts fixed reflected their short duration of growth and low biomass accumulation (frequently <3 t shoots dry matter ha^-1^) rather than poor %Ndfa (global averages 55–58%; Peoples et al., 2021). The highest inputs of BNF were contributed by lupin (130 kg N ha^-1^ global average) and faba bean (148 kg N ha^-1^ average), which are associated with a high reliance on BNF (global average Ndfa 70–71%; Table 9) and the accumulation of large amounts of biomass (often 7–8 t ha^-1^ shoot dry matter). It should also be noted that shoot-based estimates of BNF such as those depicted in Table 9 will inevitably underestimate total BNF inputs, as they do not account for N associated with the nodulated roots which could represent between 25% and 40% of the total plant N (Wichern et al., 2008, Fustec et al., 2010, Unkovich et al., 2010, Herridge et al., 2022). Although there are limited data, field ^15^N-enrichment studies suggest that the %Ndfa of both above-ground and below-ground legume N is similar (Carranca et al., 2015, Rymuza et al., 2020).

The relative contributions of different legume crops and geographic regions to total global inputs of BNF can be calculated from FAOSTAT (2021) regional production data and aggregated %Ndfa values from Table 9 with the use of algorithms to convert grain production into total (i.e., above- + below-ground) legume biomass N (Peoples et al., 2021, Herridge et al., 2022). Of the 34.4 Tg N calculated to be fixed globally by all grain legumes in 2019 using this approach, South America provided the largest BNF inputs, equivalent to 42% of the total (predominantly by soybean: 13.8 Tg N). This was followed by Asia which represented 23% of the total via pulses (3.4 Tg N), soybean (2.8 Tg N) and groundnut (1.7 Tg N); North America contributed 21% (mostly by soybean: 6.4 Tg N), Africa 10% (pulses 1.6 Tg N and groundnut 1.5 Tg N), Europe 4% (both soybean and pulses 0.6 Tg N each) and Oceania 1% (0. 2 Tg N from pulses).

Comparable BNF data reported for legume green manures and cover crops suggest %Ndfa can be expected to be 60–85%, with the amounts of N fixed frequently representing 80–150 kg shoot N fixed ha ^-1^ per crop or year across a range of environments (Giller, 2001; Mueller and Thorup-Kristensen, 2001; Li et al., 2015; Peoples et al., 2017). Similarly, high %Ndfa values are also characteristic of legumes in grazed pastures and intensive forage systems, although the amounts of N fixed are heavily dependent upon the legume composition of the forage swards and will be influenced by whether the legume is an annual or perennial species (Peoples and Baldock, 2001, Carlsson and Huss-Danell, 2003, Peoples et al., 2012). There are no global databases equivalent to FAOSTAT that can provide comprehensive information on the areas of land under legume green manures, cover crops and forages or their production; however, the annual amounts of N_2_ fixed by legumes in Australian pastures have been estimated to represent ~4 Tg N (Peoples et al., 2012).

### 4.5 Contributions of N_2_-fixing systems to soil N dynamics

Wheat, rice and maize are usually grown in cropping sequences which include not only a diverse range of other cereals (e.g., barley, *Hordeum vulgar*; oats, *Avena sativa*; sorghum, *Sorghum bicolor*) but also non-legume oilseeds (e.g., canola/rapeseed, *Brassica napus*; mustard, *B. juncea*; sunflower, *Helianthus annuus*) in addition to legumes. Some of the alternative cereals and all the non-cereals contribute to multiple (mostly beneficial) effects on growth and yield (Angus et al., 2015). In the case of legumes these benefits include both N and non-N effects (Chalk, 1998a, Angus et al., 2015). Non-N effects (such as disrupting the cycles of pests and diseases, weed suppression, residual soil moisture, changes in various soil structural and chemical properties or shifts in the composition and population size of soil microbial and invertebrate communities) are beyond the scope of this paper, and readers are referred to other reviews for further details (e.g., Bullock, 1992; Peoples et al., 2009a; Angus et al., 2015; Watson et al., 2017; Xue et al., 2016; Stagnari et al., 2017; Smith and Chalk, 2020). The potential sources of N benefits which cereals derive from BNF and N_2_-fixing systems from their effects on the supply of soil mineral (inorganic) N, and the replenishment of SON will be the focus of discussion in the following sections.

#### 4.5.1 Impact on soil mineral N

There are numerous reports of increased size in the pools of available soil mineral N for cereals following the cultivation of legumes compared to non-legumes (e.g., Peoples et al., 1995a; Ladha et al., 1996; Fillery, 2001; Ladha et al., 2000; Fustec et al., 2010; Angus et al., 2015; Plaza-Bonilla et al., 2017; Franke et al., 2018). While in-crop mineralization is important for cereal N nutrition (Peoples et al., 2017), the widely observed improvements in N uptake, growth and grain yield of cereals grown immediately after legumes are generally attributed to the additional concentrations of soil mineral N accumulated during the fallow period prior to sowing (George et al., 1995, Ladha et al., 1996, Angus et al., 2015).

Improvements in soil N availability after legumes can arise from several different sources. These include: (a) residual carry-over of mineral N unutilized by the legume during the growing season (‘nitrate-sparing’; Herridge et al., 1995; Ladha et al., 1996), (b) ‘pool-substitution’ of legume-derived N for SON (George et al., 1995, Peoples et al., 2009a); (c) lower microbial immobilization of N from legume residues than cereal stubble (Green and Blackmer, 1995), or (d) digestion and excretion of N by animals grazing N-rich legume foliage (Ledgard, 2001, Peoples and Baldock, 2001). However, the dominant pathway for biologically fixed N in legume tissue to enter the soil N pool is generally considered to occur as a component of the total legume organic residue N which becomes available as the result of decomposition and the N mineralization and immobilization processes (Chalk, 1998b, Kumar and Goh, 2000). Often mineralization studies of legume residues have primarily considered shoot and leaf materials, but organic N associated with nodulated roots can also be an important source of fixed N contributing to pools of soil mineral N acquired by following legume grain crops, cover crops and grazed pastures (Chalk et al., 2002, Peoples et al., 2009a, Peoples et al., 2012, Li et al., 2015). Indeed, the available data suggest that about 30% of the N in the stubbles of grain legumes and 20% of the N in nodulated roots may mineralize in the year following a grain legume crop, and that a subsequent wheat crop recovers on average about 20% of the grain legume residue N remaining in above-ground stubble and about 10% of the N in the below-ground plant N (Evans et al., 2001).

Decomposition of organic residues typically follow a characteristic pattern, with an initial rapid decline followed by a period of slow decrease (Fillery, 2001). The magnitude and timing of the release of legume N as plant-available forms represents a balance between the microbial-mediated mineralization and immobilization processes in the soil, which in turn are affected by the efficiency of use of the legume organic C by the decomposer population and the microbial demand for C and N for growth (Kumar and Goh, 2000, Fillery, 2001). Inorganic N tends to be released from plant residues once excess C has been consumed by microbial growth (Williams et al., 2017). Apart from the location of the legume residues (e.g. as standing stubble, on the soil surface, or incorporated into the soil) and climatic conditions (especially impacts on soil moisture and temperature to stimulate microbial activity), the main factors considered to influence mineralization and immobilization are the chemical composition of the above- and below-ground legume residues, including the N concentration, C:N ratio, cellulose, lignin and/or polyphenol contents, and size of the soluble C and N fraction (Giller and Cadisch, 1997; Clement et al., 1998; Kumar and Goh, 2000; Bolger et al., 2003; Peoples et al., 2004a). These compositional features of residues can vary across legume species but will also be dependent upon whether the residues are young or from mature plant materials (Kumar and Goh, 2000). For example, the C:N ratio of green manured and cover-cropped legumes is commonly < 20:1 which is conducive to net mineralization in the short- to medium-term (Kumar and Goh, 2000, Thorup-Kristensen et al., 2003). By contrast, the senesced vegetative materials remaining after grain legume harvest are often > 30 (Peoples et al., 2017) and can induce transient net immobilization. However, since the C:N ratio of stubble of harvested cereal crops tend to be much greater (75–160:1), the duration of net immobilization will be considerably longer. Both the higher N content and lower C:N ratios of legume residues (regardless of whether these are green manure or senesced materials) than the stubble of cereals and other non-legumes crops will result in greater net N mineralization, providing an excess of mineral N with respect to microbial growth and resulting in higher soil mineral N concentrations (Clement et al., 1998, Kumar and Goh, 2000, Thorup-Kristensen et al., 2003, Plaza-Bonilla et al., 2017, Williams et al., 2017).

Information generated from 16 rainfed wheat cropping systems experiments undertaken in south-eastern Australia indicated that the increased available soil N observed immediately prior to sowing wheat in the following growing season after 26 different legume crops grown through to maturity and harvested for grain or five legume brown manure treatments represented on average 35 and 60 kg of additional mineral N ha^-1^; respectively compared to after either wheat or canola (Peoples et al., 2017). In studies where the experimentation was continued into a third year of the cropping sequence significantly higher soil mineral N were still detected before sowing the second wheat crop following one-third of the legume grain crops (representing 18 kg additional mineral N ha^-1^ on average) and all the brown manured legumes (26 kg mineral N ha^-1^ on average; unpublished data). The extra mineral N at the beginning of the second and third growing seasons was calculated to be equivalent to 28% and 10%; respectively of the total legume N estimated to be remaining in the combined above- and below-ground residues of the first-year legume pulse crops, and 24% and 11% of the total legume residue N from the first-year brown manures. Based on the measured increases in wheat total N uptake over the two wheat cropping cycles, the apparent recovery of N from the preceding legume grain crops and brown manures represented 29%− 30% of the legume residue N by the first wheat crop, and around 5% by the second wheat (Peoples et al., 2017; unpublished data). Given the relatively low yielding environment for rainfed wheat grain production in Australia (2.02 t ha^-1^ mean 2015–19; FAOSTAT, 2021) and the low rates of fertilizer N routinely applied (44 kg N ha^-1^ on average to the 12.6 Mha of wheat grown in 2014: Heffer et al., 2017; FAOSTAT, 2021), the observed improvements in available soil N and wheat N uptake following legume cropping represents substantial potential savings in fertilizer N. However, based on previous observations from farming systems research undertaken in Australia and elsewhere in the word, the impact of a single year of legume cropping would not be expected beyond the second successive wheat crop (Chalk, 1998b, Fillery, 2001, Giller, 2001, Angus et al., 2015).

In the case of wheat following 3–4 years of self-regenerating annual clover or alfalfa-based pastures, the concentrations of plant-available soil N observed at the start of the cropping phase can frequently be in the range of100–200 kg N ha^-1^, although values of up to 300–400 kg N ha^-1^ have been detected after highly productive pure legume swards (Angus et al., 2000, Fillery, 2001, Peoples and Baldock, 2001, Peoples et al., 2004a). On average the increased concentrations of soil mineral N after forage legume measured above that following either a bare fallow or pure grass sward were equivalent to 14–15 kg additional mineral N ha^-1^ per t of above-ground legume dry matter grown during the pasture phase (Peoples et al., 2004a, Angus and Peoples, 2012), although this can also be mediated by the intensity of grazing during the pasture phase, when the pasture sward is terminated, either by plowing or with herbicide prior to cropping and the amount of rainfall during the fallow period (Angus et al., 2000, Ledgard, 2001, Fillery, 2001, Peoples and Baldock, 2001). The general pattern of soil N release and improvements in cereal N uptake tend to be similar after a legume-based pasture than after grain legumes. Data derived from Australian and North American field studies suggest that the first wheat or maize crop grown following an annual forage legume or alfalfa might recover the equivalent of 17%− 25% of the legume N with the second cereal crop recovering a further 1%− 4% (Harris and Hesterman, 1990, Angus and Peoples, 2012). However, there can be a lag in availability of soil N after perennial legumes, such as alfalfa in rainfed environments, due to drier soil profiles and initial net N immobilization of N derived from alfalfa roots (Peoples and Baldock, 2001; Bolger et a, 2003; Angus and Peoples, 2012). The other main difference is the N benefit can often persist beyond the second cereal crop because of the multiple years of BNF inputs by forage legumes and the large pools of below-ground organic legume N present, although net mineralization rates would be similar to the native SON of around 2% per year (Wichern et al., 2008, Angus et al., 2000, Angus and Peoples, 2012, Peoples et al., 2012).

Another example of the impact of legumes on soil N dynamics comes from experimentation with the use of legume cover-crops in irrigated maize production in central Spain (vetch with or without barley cover-crop in a maize–sunflower–cover crop–maize rotation) and the Mid-Atlantic region of the USA (vetch, pea or clover cover crops in a maize–cover crop–soybean–winter wheat–cover crop–maize rotation), reviewed by Kaye and Quemada (2017). In both case study regions, autumn-sown legume cover crops accumulated between 50 and 300 kg N ha^-1^ in above-ground biomass before termination prior to maize crops in spring. The amount of cover-crop N mineralized to supply N for the cash crop was generally equivalent to between 33% and 50% of the cover-crop N when residues were not incorporated. Consequently, maize grown after legumes in these regions would be expected to require 20–150 kg N ha^-1^ less fertilizer than maize without a cover crop, which is consistent with previously reported “fertilizer replacement” values of cover crops (Kaye and Quemada, 2017).

It has been speculated that one further pathway for legumes to contribute to the N nutrition of cereals in intercropping and mixed cropping systems could be via the direct transfer of fixed N to neighboring cereals during the growing season (Homulle et al., 2021). It is often assumed that any legume N mineralized from senesced fine roots, nodules or fallen leaves, or N released into the legume rhizosphere over the growing season will be predominantly captured by nearby cereal roots (see Peoples et al., 2015; Homulle et al., 2021 for further details of proposed mechanisms). It is an extremely challenging task to demonstrate conclusively such direct transfer of N from legume to companion cereal or to quantify its flux (Chalk et al., 2014, Peoples et al., 2015). However, most reports suggest that the total N benefit derived by a cereal from an intercropped legume (only a portion of which would originate from BNF) within a growing season might represent < 10 kg N ha^-1^ (Chalk, 1996, Peoples et al., 2015). It is more likely that the additional N uptake observed in intercropped cereals than in sole cereal crops reflects the greater access and assimilation of plant-available N by each cereal plant in intercrops. This is because the intraspecific root competition between two neighboring cereal plants for available water and soil N in a pure cereal crop is greater than between a cereal and legume in an intercrop mix (Giller, 2001, Peoples et al., 2009a, Jensen et al., 2020). The higher N use efficiency and more effective scavenging of soil mineral N by the cereal component of the intercrop is supported by the many reports of higher %Ndfa by intercropped legumes compared to where legumes were grown in pure stands (e.g., Rerkasem et al., 1988; Giller, 2001; Bedoussac et al., 2015; Watson et al., 2017; Jensen et al., 2020).

#### 4.5.2 Impact on the soil organic N pool

Nitrogen supplied from SON remains the principal source of N to support cereal crop growth, despite increasing use of synthetic fertilizer N produced from the Haber-Bosch process (Broadbent, 1984). Numerous short- and long-term research trials carried out globally over the last five decades suggested that on an average, wheat, rice and maize obtained 48% of their N from fertilizer and 52% of N from soil sources (Ladha et al., 2016). If the native soil organic matter provided the bulk of this N, the soil N reserves would be expected to be progressively depleted over time (Brye et al., 2003, Crews and Peoples, 2005). Yet a meta-analysis of measured changes in SON in 114 long-term continuous cereal experiments conducted globally did not indicate the anticipated extent of decline (Ladha et al., 2011). Instead, the soils cultivated with cereals seemed to have approached a more-or-less steady state, suggesting that in addition to fertilizer N, there were other sources of N inputs provided to cereal-based cropping systems that contributed to replenishing much of the soil N either lost or removed in harvested products. Such other sources of N could include inputs of N via (a) farm-yard manure, (b) the planted seed, (c) recycling of N from above-ground crop residues, (d) atmospheric N deposition (via both rain and dust), and (e) non-symbiotic BNF in soil and plant systems. After considering the estimates of contributions of N from all other potential sources, the unexplained balance representing 24.6% of N removed by cereals was attributed to non-symbiotic BNF (Ladha et al., 2016). These calculations implied that BNF played a key role in maintaining the observed N equilibrium in the soil N pool under continuous cereal cultivation and constant crop management.

**Fig. 1:**
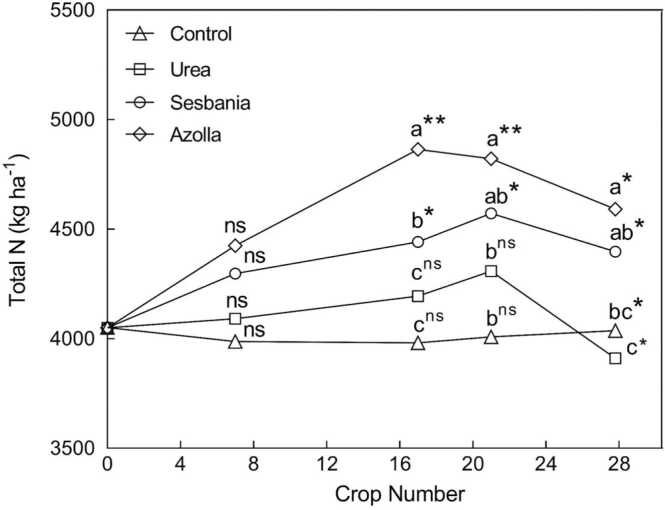
Trends in measures of total soil N (to a depth of 0.5 m) in a long-term continuous rice-rice system experiment at the IRRI farm, Philippines comparing a nil-N treatment with the inputs of synthetic N (137 kg N ha^-1^ per year), and the inclusion of sources of BNF via azolla or sesbania (adapted from Ladha et al., 2000). Different letters shown between treatments indicate statistical significance (α =0.05) according to Duncan’s multiple range test. * and ** indicate significance at the 0.05 and 0.01 levels, respectively

In situations where legumes are grown either as short-term cover crops or for the purpose of providing sources of green and brown manure production, all fixed N in the organic matter will be returned so there will be a direct contribution to SON (Clement et al., 1998; Li et al., 2015; Maseko et al., 2020; Zalak and Parthsing, 2021). For example, Ladha et al. (2000) examined the long-term effects of N fertilizer from different sources (*Azolla* and *S. rostrata* grown in situ, or urea) on N balances, soil N pools and grain yields in a 14-yr double-crop rice rotation. After 27 crops, the cumulative positive N balance was estimated at 1.24, 0.35, 0.65, and 1.04 t N ha^-1^ in the control (without N), urea, sesbania and azolla plots, respectively. Total soil N (0–0.5 m soil depth) gradually increased with time and reached 344–541 kg N ha^-1^ after 14 years in the *Sesbania* and *Azolla* treatments (Fig. 1 and Table 10). This means that despite the high amounts of N removed with the rice grain and straw, the soil N status was conserved due to a net positive N balance, partially reflecting N contributed from non-symbiotic N_2_ fixation (13–46 kg ha^-1^ crop^-1^), and symbiotic N_2_ fixation (57–64 kg N ha^-1^ crop^-1^) by *Sesbania* and *Azolla*, which resulted in rice responses equivalent to applications of 60 kg of fertilizer-N ha^-1^ as urea (Table 10).

**Table 10:** Cumulative N balance sheet for lowland rice soil at 0.5 m depth after 27 continuous rice crops. IRRI field, 1985 wet season to 1998 dry season^a^.

Fertilizer treatment	Crop (A)	Change in soil N (B)	Fertilizer input (C)	Other inputs^b^ (D)	N balance (A+B) – (C+D)		Grain yield (t ha^-1^)	
					Total	Per crop^c^	Total	Per crop
	-----------------------------------------------kg ha^-1^-------------------------------------------------------							
Control	1656c^d^	-8bc	0	405	1244a^e^	46	111.1a	4.1a
Urea	2598b	-134c	1710	405	348b^e^	13	152.1b	5.6b
Sesbania	2707ab	344ab	2001	405	646b^e^	24	149.4b	5.5b
*Azolla*	2781a	541a	1782	501	1039a^e^	38	156.0b	5.7b
SE^f^	37.1	111.9			111.4			

a Adapted from Ladha et al. (2000).

b Other inputs include N from rain and irrigation water, P fertilizer, pesticides, etc.

c N gains per crop.

d Means in a column followed by a different letter are significantly different at the 0.05 probability level by Duncan’s Multiple Range Test (DMRT).

e significantly different from zero at the 0.05 and 0.01 probability level, respectively

f Standard error

**Table 11:** Average global estimates of sources of N (kg N ha ^-1^) in crop harvest of continuous maize, rice, and wheat production systems for 50 years and measured N removed in grain (1961–2010). Data in parentheses represent the percentages of the total N removed by crop^a^.

Crop	Fertilizer	Soil N reserve	Manure	Atmospheric deposition	Crop residues	Seed	Calculated non-symbiotic BNF	Total N removed in grain	Mean grain yield (t ha^-1^ yr^-1^)
Maize	44.4	4.9	9.9	4.7	1.4	0.3	12.7	78.3	3.6
Rice	25.8	-3.6	11.3	3	0.9	0.2	22.4	60.1	3.2
Wheat	24.9	5.6	6.5	4.1	0.8	1.2	12.7	55.7	2.1
Mean	31.7	2.3	9.2	3.9	1	0.6	15.9	64.7	
	(48.1%)	(4.4%)	(14.0%)	(6.2%)	(1.6%)	(1.1%)	(24.6%)	(100%)	

a Adapted from Ladha et al. (2016).

However, in the case of grain legumes, the amounts of N_2_ fixed will not always be sufficient to ensure a net input of fixed N to contribute to SON. This is because a considerable portion of the N accumulated by a grain legume is removed from the field in grain (Myers and Wood, 1987, Peoples et al., 2009a, Ciampitti and Salvagiotti, 2018). For grain legumes to play a positive role in the maintenance of soil N fertility, they must leave behind more fixed N in residues than the amounts of soil N in the harvested grain, otherwise there will be a net depletion of the soil N pool. This will be regulated by %Ndfa and the quantity of the legume N accumulated over the growing season partitioned in grain, as defined by a crop’s N harvest index (NHI; Myers and Wood, 1987; Chalk, 1998b). The NHI provides a measure of grain N expressed as a fraction (or percentage) of total above-ground biomass N at maturity (i.e., the sum of N contained in grain, pods, stems, petioles, and attached and fallen senesced leaves). Determinations of NHI vary with legume species and are dependent upon the N content of the vegetative residues, as well as grain yield and N content. Because of its high grain protein content and yield, soybean has a higher NHI (0.7–0.8; Salvagiotti et al., 2008, except at grain yields <2 t ha^-1^; Herridge et al., 2022) than most other grain legume species (typically NHI of 0.4–0.6; Peoples and Craswell, 1992; Giller, 2001; Walley et al., 2007).

It is necessary for %Ndfa to exceed NHI to achieve a net input of fixed N for SON (Myers and Wood, 1987, Peoples and Craswell, 1992). Two comprehensive reviews of soybean N balance have been undertaken using published research data generated across a range of N fertilizer treatments from 1966 to 2006 (*n* = 321; Salvagiotti et al., 2008) and 1955–2016 (*n* = 460; Ciampitti and Salvagiotti, 2018). These two studies calculated soybean NHI as 0.73 and average %Ndfa values of 52% and 55%, respectively. The analyses revealed that a net positive balance occurred in only 17–20% of trials, and in keeping with soybean’s NHI being higher than average %Ndfa, the mean N balance determined across all experiments and treatments was found to be strongly negative for both datasets (−40 and −47 kg N ha^-1^ based on above-ground N). However, BNF inputs were calculated to be closer to balancing grain N removal (N balances of −4 and −13 kg N ha^-1^, respectively) if BNF estimates were adjusted to include additional contributions of N associated with soybean’s nodulated roots, by assuming 24% of the total plant N was below-ground (Rochester et al., 1998).

Comparable N balance studies have only been undertaken for a limited number of other grain legume species. These include experimental data from rainfed and irrigated chickpea (*n* = 82), field pea (*n* = 79), lentil (*n* = 38), common bean (*n* = 31) and faba bean (*n* = 10) crops in the Canadian Northern Great Plains (Walley et al., 2007). With NHI ranging from 0.47 (pea) to 0.65 (faba bean) and mean %Ndfa between 41% (common bean) and 84% (faba bean), analyses of the dataset indicated that all faba bean crops and > 50% field pea and lentil were calculated to have positive N balances. Chickpea and common bean on the other hand typically fixed less N than harvested in grain even when calculations included additional N contributed by nodulated roots (assuming 14% crop N remaining in root residues and a further 10% in rhizodeposition; Walley et al., 2007). The other major review of legume N balance was undertaken for data collated from Australian experimental and commercial rainfed crops, which included chickpea (*n* = 82), field pea (*n* = 118), faba bean (*n* = 36), and lupin (*n* = 26) (Evans et al., 2001). This particular study concluded that around 85% of Australian crops had a net positive contribution, with the average differences between total fixed N (i.e., above + below-ground N) and N harvested representing + 6 kg N ha^-1^ for chickpea, + 40 kg N ha^-1^ for field pea, + 80 kg N ha^-1^ for lupin and + 113 kg N ha^-1^ for faba bean (contributions of fixed N from the nodulated roots assumed to represent 42% of total plant N below-ground for chickpea, pea 20%, lupin 28% and faba bean 43%; Unkovich et al., 2010).

All four N balance reviews reported large variations in the derived N balances. Evans et al. (2001) highlighted the influence of rainfall and different geographic location on the calculations, but a recent analysis of annual N balances of cool-season pulses by Smith and Chalk (2020) also identified other factors that influenced short-term temporal and spatial variability in %Ndfa and/or legume growth. These included concentrations of soil mineral N at sowing, legume cultivar and sowing date, and the management of non-legume crops and their residues in the preceding cropping season. However, what is clear is that some grain legumes are more likely to result in positive N balances and provide agronomically useful net inputs of fixed N to SON (e.g., field pea, lupin, faba bean) than others (e.g., soybean, common beans, chickpea).

All the above calculations of N balance and the resulting conclusions assumed full retention of vegetative residues after grain harvest. It is worth noting that grain legume residues are not always returned to the soil. For example, in some South and West Asian farming systems animals either graze the standing legume stubble following grain harvest or all above-ground crop biomass is removed at maturity for off-site grain threshing, with the legume haulms subsequently fed to livestock (Beck et al., 1991, Hobbs and Osmanzai, 2011). In both cases, the dung generated by livestock may also be collected and dried for fuel. Consequently, under these farmer practices no vegetative organic matter is returned to the land and the net N balance will always be negative, as the fixed N that remains in situ associated with any remaining nodulated roots will be insufficient to compensate for the removal of both the shoot and grain N (Beck et al., 1991).

## 5 Ecological intensification in cereal-based farming systems

Synthetic N revolutionized cereal production by enhancing crop growth and grain yield, and increasing the area and time available for cereal cropping, as it eliminated the need to specifically allocate land for soil fertility rejuvenation during crop rotation (Smil, 2001). This contributed significantly to an increase in global food production (Crews and Peoples, 2004). The use of synthetic N in cereal production increased exponentially during the second half of the twentieth century and now represents around 50–60 Tg N year^-1^ (Heffer et al., 2017, Ladha et al., 2020). In many regions, fertilizer costs are low, and their liberal or excessive use has led to distinct environmental and ecological degradation problems, including depletion of soil organic matter due to oxidation of soil C, surface and groundwater pollution, and increased emissions of greenhouse gases (Peoples et al., 2004b, UNEP and WHRC, 2007, Erismann et al., 2008). While synthetic N will undoubtedly remain essential for ensuring global food supply, and optimizing the efficiency of its use remains a key objective (Crews and Peoples, 2005, Ladha et al., 2005, Ladha et al., 2020), it is clear that it is also desirable to explore strategies to increase inputs from alternative sources of N with lower environmental costs – such as BNF – to provide opportunities to reduce agriculture’s high reliance upon fertilizer N (Jensen et al., 2012, Ladha et al., 2016, Peoples et al., 2019, Udvardi et al., 2021).

### 5.1 Opportunities to improve BNF contributions

Numerous studies compiled in this review have demonstrated that improvements of BNF are possible with well-targeted management practices and appropriate genetics (SI Table 2, Table 3, Table 4, Table 5, Table 8, Table 9). This suggests that in principle there are prospects of further enhancing inputs of BNF. Based on a review of the international literature, our own knowledge, and expert opinion and advice, Table 12 has been developed to provide an assessment of the potential achievable levels of BNF, the relative advantages and constraints, and the outlook for various conventional non-symbiotic and symbiotic systems. While a theoretical upper limit of 60–80 kg N ha^-1^ crop^-1^ has been proposed for non-symbiotic N_2_ fixation in cereals by assuming there is an abundant population of diazotrophs in and around the plant and unlimited pools of available C, such high values are unlikely to be attainable. The BNF potentials listed for aquatic green manures (260 kg N ha^-1^ crop^-1^) and grain legumes (245–290 kg N ha^-1^ crop^-1^; Table 12) on the other hand are not unprecedented or unrealistic, as comparable values have already been reported in the literature. Table 13 lists agronomic practices considered likely to favor improved BNF, either directly or indirectly. The following sections consider how the options provided in Table 13 and the application of simulation modeling and other decision-support tools could aid the identification of the specific management or genetic opportunities and land-use strategies with the potential to improve BNF inputs in the broad framework of sustainable intensification.

**Table 12:** Commonly reported range in estimates of BNF (kg N ha^-1^ crop^-1^), proposed potential levels achievable, and the prospective outlook for improving BNF inputs from various N_2_-fixing systems^a^.

BNF system	Common range of reported BNF	Theoretical maximum BNF potential			Advantage	Constraint	Status of adoption by farmers	Outlook
		Proposed	Assumptions					
Total non-symbiotic N_2_ fixation	rice: 18–51 wheat: 3–40 maize: 13–26	60–80 in rice, wheat, and maize		Prolific populations of endophytic and rhizospheric N_2_-fixing bacteria All C input (2 t crop^-1^) is used by N_2_ fixers 40 mg N is fixed g C^-1^	Inherent to the system	Prone to N loss Improvement is difficult	Widely used by default	Potential to improve through agronomic (including straw) management as part of soil health agenda
Cyanobacteria in rice cultivation	0–80	70		Photosynthetic aquatic biomass is composed of exclusively of N_2_-fixing BGA (C:N = 7) Primary production is 0.5 t C ha^-1^ crop^-1^	Inherent to the system	Requires continuous standing water Inhibited by combine N in flood water Grazer inhibits growth of cyanobacteria	Widely used by default	Low potential because of difficulty in managing the algal bloom as inoculations do not work
*Azolla* in rice cultivation	20–150	225		Two *Azolla* crops grown and incorporated per rice crop	High (>80%) %Ndfa and large amounts of N produced Improves SOM Reduces N volatilization loss Reduces weed pressure	Requires continuous standing water on soil surface Labor intensive Difficult in maintaining inoculum supply	Use by the farmers has declined, and currently negligible	Low or negligible potential
Aquatic legume green manure in rice cultivation	20–260	260 in 55 days		Fast-growing species such as *Sesbania rostrata* is used as green manure	High %Ndfa (80–90%) and large amount of N production Improves soil organic matter	Farmers prefer legumes with economic value Labor Intensive	Use by the farmers declined, currently insignificant use	Modest potential in single rice cropping system in Africa and some parts of Asia
Grain legumes in cereal rotations	57 kg total N fixed ha^-1^ (common bean) to 212 kg total N fixed ha^-1^ (faba bean)^b^	245–290 kg total N fixed ha^-1^		Legume crops other than common bean 10–12 t shoot dry matter ha^-1^ (3.5–4 t grain ha^-1^) %Ndfa of 85%, 20 kg N fixed per t shoot dry matter accumulated Nodulated roots represent 30% of total crop N	Inherent to the system Provide multiple rotational benefits that improve cereal productivity	Dominant cereals restrict legume cultivation Cereals have larger markets and are easier to grow than legumes Grain economic value is highly volatile Many pulses are susceptible to disease and insect pests	Widely adopted by the farmers, but their inclusion in farming systems driven by fluctuations in market demand and value	High potential to enhance yield and improve consistency of legume productivity through agronomic management and breeding

a Adapted from Herridge and Bergersen (1988), Chalk (1991), Roger and Ladha (1992), Ladha and Reddy (1995), Peoples et al. (1995a), Gupta et al. (2006), Roper and Gupta (2016).

b Global average amounts of shoot N fixed presented for common bean and faba bean in Table 9 adjusted to include assumed below-ground contributions of fixed N associated with nodulated roots represented ~30% of total plant fixed N (Herridge et al., 2022).

**Table 13:** Agronomic practices with potential to enhance BNF inputs in cereal-based farming systems.

Practice	Likely mechanism for enhancement of BNF inputs	References
Zero or reduced tillage	Positive changes in diversity and heterogeneity of rhizosphere diazotrohphic community Higher organic matter and substrate inputs in rhizosphere Lower soil nitrate from reduced disturbance of soil organic matter reduces risk of inhibition of BNF	Li et al. (2021) Zhou et al. (2020) Peoples et al., 1995b, Torabian et al., 2019
Crop residue retention	Availability of a wide range of C compounds as source of C and energy substrates by diazotrophs Residue mulch creates conducive microenvironment (i.e., moisture conservation, lower O_2_ environment, steady supply of C) for diazotrophs Crop residue of high C:N (i.e., cereal straw) immobilizes inorganic N result in stimulation of BNF	Roper and Ladha (1995) Fan et al. (2020) Palm et al. (2014)
Smart synthetic N management	Optimal rate and timely application of synthetic N to cereals improves N use efficiency and reduces risk of unutilized fertilizer N inhibiting BNF by diazotrophs during cereal phase	Ladha et al., 1998
Application of biochar	Biochar enriches soil and stores organic C in a form that provides C and energy source for diazotrophs Biochar immobilizes inorganic N so BNF less likely to be suppressed Increases P bioavailability which stimulates BNF	Laird (2008) Nelson et al. (2011) Thies and Rilling (2009)
Use of manure with or without inorganic fertilizer.	Enhances soil C storage and nutrient availability after decomposition which will serve as C and energy source for diazotrophs Supports more diverse soil microbial communities and increases microbial biomass contributing to increase in BNF	Ladha et al. (2011)
Increased water availability Controlled water application	Drought suppresses BNF process Adequate plant-available water via rainfall or irrigation increases BNF by stimulating plant growth and microbial activity ‘Saturated soil culture’ (long-term flooding) enhances nodulation and BNF by soybean	Peoples et al., 1995b, Santachiara et al., 2019
Integration of legume in fallow or in rotation as part of diversification and intensification	Increased frequency of use of legumes in cropping system results in increased inputs of BNF Supply of in situ high quality residues with high N concentration and a low C:N ratio improves soil N status	Franke et al. (2018)
Green or brown legume manure^a^	Increased frequency of use of legumes in cropping system results in increased inputs of BNF Supply of in situ legume residue with high N concentration and a low C:N ratio improves soil N status Green manure mulch and brown manuring assist the management of weeds	Becker et al., 1995, Singh et al., 2009 Peoples et al. (2017)
Intercropping legumes within cereals	Increased frequency of use of legumes in cropping system results in increased inputs of BNF Intercropped legume has higher %Ndfa than legume sole crop Increased yield stability and yield per unit area, reduced pest problems and lower requirements for agrochemicals and N fertilizer to support cereal yield	Lithourgidis et al. (2011) Bedoussac et al. (2015) Fletcher et al. (2016) Jensen et al. (2020)

a Green manure = slashing/mulching live legume crop; Brown manure = legume crop killed with knock-down herbicide prior to see-filling (an emerging farmer practice to manage herbicide-resistent weeds and improve soil mineral N for following cereal crop).

#### 5.1.1 Free-living and non-symbiotic N_2_-fixing systems

Much of the past research effort in the case of non-symbiotic N_2_ fixation occurring close to the plant (associative N_2_ fixation) or in bulk soil/flood water, has been limited to the characterization of diazotrophs, and measurements and assessment of its maximal potential. Although numerous bacterial and cyanobacterial inoculation trials have been conducted, and inoculants have been commercialized in many countries (Roger, 1996, Soumare et al., 2020), as far as we are aware few studies have observed consistent results or conclusively demonstrated the successful manipulation of non-symbiotic N_2_ fixation inputs in cereals with conventional inoculation technologies under field conditions (Giller, 2001, Chalk, 2016). Experience with rhizobial inoculants for legumes has demonstrated that a range of diverse factors (i.e., edaphic, biotic, climatic) can limit the effectiveness of inoculation, but that poor inoculum production, storage and/or application practices can also be responsible for many inoculation failures and inconsistent results (Brockwell et al., 1995, Herrmann and Lesueur, 2013, Callaghan, 2016). The same challenges face the establishment and survival of sufficient populations of any new inoculant diazotroph species or strains within the existing soil microbial community that might be necessary to elicit an inoculation response. This has prompted researchers to employ various approaches to enhancing the prospects for improved BNF and crop yields by engineering the plant-associated microbiome. For instance, diazotrophs isolated from wheat and maize (*Azotobacter chroococcum, Azorhizobium caulinodans, Rhizobium* sp*. Pseudomonas protegens, Kosakonia sacchari*) with inducible nitrogenase activity were developed through reprogramming the genetic regulation of N_2_ fixation and assimilation (Bageshwar et al., 2017, Bloch et al., 2020, Ryu et al., 2020). These strains lacked NH_3_ repression capability and were able to produce NH_3_ via N_2_ fixation in the presence of fertilizer N. In a field trial conducted in Puerto Rico, genetically modified strains (*Kosakonia sacchari* PBC6.1 and its derivatives) were reported to increase maize yield of about 1.0–1.5 t ha^-1^ compared to that of the uninoculated control which produced 6.3 t ha^-1^ (Bloch et al., 2020). Another small-scale field experiment carried out in India observed an average yield increase of 60% in wheat after inoculation with a genetically modified strain (*Azotobacter chroococcum* CBD15*)* without fertilizer N, and yield could subsequently be maintained with a saving in fertilizer inputs equivalent to 40 kg N ha^-1^ (Bageshwar et al., 2017). While both studies showed strong colonization and *nif* expression, the inoculation field results were based on a single season and further studies are required to demonstrate repeatablility of response. Furthermore, although *nif* genes were expressed, no data were presented on the amounts of N_2_ fixed. It is critical that carefully designed field trials are conducted at multiple locations and seasons to examine the impact of G × M × E interactions and undertake complementary ^15^N_2_ feeding experiments, not only to confirm BNF and quantify the amounts of N_2_ fixed, but also to track the fate of the fixed N to verify BNF contributed directly to crop nutrition and responsible for improvements in growth and yield (Giller et al., 1984, Ma et al., 2019a). Ryu et al. (2020) maintained that while there has been good progress towards building efficient strains, additional genetic engineering would be required to (a) maximize the ability of the microorganism to catabolize C sources from the plant, (b) increase the flux of fixed N delivery by redirecting metabolism, and (c) introduce transporters and the optimization of electron transfer. They also proposed the possibility of genetically engineering plants to produce orthogonal C sources such as opines or less common sugars, and then placing the corresponding catabolism pathways into the bacterium to create a synthetic symbiosis and to provide a selective niche to reduce competition by the existing soil microbial community.

Several studies of soils under cereal-based cropping sequences have utilized genetic profiling (*nif* gene sequencing analysis) to investigate how fertilization, tillage, crop rotations or other management practices affect N_2_-fixing bacterial communities (e.g., Wakelin et al., 2010; Collavino et al., 2014; Feng et al., 2018; Schmidt et al., 2019). One of the standout findings has been that reduced tillage and stubble retention result in a higher relative abundance of keystone taxa of diazotrophs (Gupta et al., 2019, Li et al., 2021; Table 13). The reduction of tillage and maintenance of crop residues on the soil surface is believed to increase biological activity by moderating soil moisture and temperature in the short-term, and by improving soil tilth and increasing organic matter content over the longer term (Palm et al., 2014). Reduced tillage practices also result in lower concentrations of soil NO_3_^-^ which minimizes the inhibition of BNF; a decreased level of soil disturbance is also conducive for the generation of soil pore networks by which stubble decomposing organisms and N_2_-fixing bacteria can interact. This increases the number of soil microsites with available C and enhances the formation of macro-aggregates critical for the development and maintenance of reduced O_2_ tension required for N_2_ fixation by many free-living diazotrophs (Gupta et al., 2019). Residue mulch also encourages a favorable micro-environment for diazotrophs by assisting moisture conservation, generating a lower O_2_ environment and providing a ready supply of C (Roper and Ladha, 1995; Table 13).

Further efforts are needed to explore the influence of soil nutrient management on free-living N_2_ fixation. One recent example where this was undertaken using a ^15^N_2_-labeling ﬁeld-based growth chamber-based method (74 days incubation; Wang et al., 2020) reported a doubling of non-symbiotic N_2_ fixation, from 22 to 53 kg N ha^−1^ through the application of molybdenum (Mo) in a rice-Inceptisol system. The application of Mo signiﬁcantly increased the number of *nifH* gene copies and the relative abundance of cyanobacteria in both growth chamber and microcosm experiments. While these findings appear promising, additional studies are required to confirm the effects of Mo application on BNF and to ascertain whether it provides improvements in cereal N uptake or subsequent soil N availability. Another area that warrants investigation is whether fertilizer N management in cereals, including timing and rates applied, plays a role in regulating BNF input. Potentially, there can be two mechanisms whereby N fertilizer could affect free-living BNF: (a) N fertilizer may augment BNF/SON by promoting plant growth, thereby increasing the amount of litter added to soil (i.e., additional C substrate for free-living diazotrophs; Glendining and Powlson, 1995), or (b) N fertilizer can inhibit BNF and increase the rate of loss of SON by accelerating the rate of oxidation or decay of litter and indigenous organic material (Mulvaney et al., 2009). We hypothesize that the latter process might be avoided and BNF further enhanced if excess use of synthetic N is circumvented along with N application timing being optimized, so that diazotrophs function according to their potential (Table 13). Biochar (a charcoal produced from crop residue) could perhaps also be applied as a complementary practice, as this has also been implicated in promoting BNF (Laird, 2008; Table 13). Biochar not only serves as a potential C and energy source for diazotrophs and provides suitable mico-sites for BNF activity, but also immobilizes inorganic N and increases phosphorus bioavailability, both of which might encourage BNF (Laird, 2008, Nelson et al., 2011, Thies and Rilling, 2009).

Crop cultivar differences in %Ndfa have been reported in all three cereals (SI Table 5), although it has not been established if the observed differences are necessarily genetically based or consistently repeatable. Indeed, misleading conclusions about reputed differences in BNF between cereal varieties have sometimes arisen due to the inherent limitations of the various methodologies commonly used to quantify BNF, or to the faulty interpretation of experimental data (Boddey, 1987, Chalk, 2016, Unkovich et al., 2020).

Large differences in root-associated *nifH-*gene expression in diazotrophic communities have been observed in cultivated and wild rice species (*Oryza brachyantha*; Knauth et al., 2005) which could represent valuable germplasm for further breeding efforts aimed at enhancing associative BNF in rice. Wu et al. (1995) attempted to map the genes underlying rice cultivar ability to stimulate non-symbiotic N_2_ fixation, and based on RFLP analysis concluded that the trait may be controlled by multiple genes. However, to our knowledge no further attempts have been made to exploit this information for breeding purposes. This raises some serious questions regarding the ability to use specific plant genotypes to stimulate to enable further enhancement, excepting possibly through genetic engineering. This will be discussed in greater detail in Section 4.2.4.

#### 5.1.2 Symbiotic systems (legumes)

To achieve the desired outcome of increased inputs of fixed N by legumes the interaction between the best legume and rhizobial genotypes tailored to the local environment and grown with the best agronomic management (i.e., G_L_ x G_R_ x E x M) needs to be understood, identified and exploited (Giller and Ronner, 2019; Vanlauwe et al., 2019). Which legume species to grow in a particular farming systems, soil type and climate, the potential impact of specific breeding and selection targets, and the necessary agronomic interventions and crop sequences that integrate the best combinations of G_L_ x G_R_ x M to improve BNF through either enhancing %Ndfa and/or legume productivity, can be facilitated through close engagement and consultation with farmers (Giller and Ronner, 2019, Peoples et al., 2019, Pelzer et al., 2020), and evaluated with the application of simulation models (e.g., Herridge et al., 2001; Grassini et al., 2015; Hochman et al., 2020; Smith and Chalk, 2020).

A priority will be the implementation of strategies to establish sufficient populations of rhizobia in the soil (compatible and effective with the chosen legume species) to ensure adequate root nodulation (>1000 rhzobia g soil^-1^); especially when a new legume crop with specific rhizobial requirements is sown for the first time, in highly acidic and alkaine soils where rhizobial persistance is anticipated to be poor, or when the gap between sowing the same legume species exceeds 6 years (Brockwell et al., 1995, Peoples et al., 2009a). Prospective agronomic practices to achieve this would include the use of high quality rhizobial inoculants at sowing, efficient inoculation practices, and the ameliorating of any soil conditions that are either hostile to rhizobia’s survival or results in erratic nodulation (e.g., soil pH or nutrient deficiencies; Brockwell et al., 1991; Brockwell et al., 1995; Peoples et al., 2009a; Giller and Ronner, 2019; O’Hara et al., 1988; Vanlauwe et al., 2019). To achieve high %Ndfa concentrations of available soil mineral N would also need to be low at sowing (<55–85 kg N ha^-1^; Voisin et al., 2002; Salvagiotti et al., 2008) because of the inhibitory effect of high concentrations of inorganic N on nodule initiation and BNF (Peoples et al., 2009a, Guinet et al., 2018, Santachiara et al., 2019). This is most likely to occur (a) under reduced tillage practices and the retention of stubble from a previous cereal crop to immobilize soil mineral N (Peoples et al., 1995b, Torabian et al., 2019), (b) when grain legumes are intercropped with a cereal (Bedoussac et al., 2015, Watson et al., 2017, Jensen et al., 2020), (c) if legumes are grown following a cereal or non-legume oilseed crop rather than after a long period of fallow (Peoples et al., 2009a, Smith and Chalk, 2020), or (d) when forage legumes are established in mixed species swards rather than being sown in pure legume pastures (Peoples and Baldock, 2001, Carlsson and Huss-Danell, 2003, Peoples et al., 2012). Potential genetic approaches to improving %Ndfa include selecting highly effective rhizobial strains, and breeding legume germplasm which are either more promiscuous in their rhizobial preferences and/or whose nodulation is more tolerant of soil NO_3_ or acidity (Giller and Cadisch, 1995, Herridge and Danso, 1995, Peoples et al., 2009a, Vanlauwe et al., 2019).

Given that close relationships have frequently been observed between legume productivity and the amounts of N_2_ fixed by many different crop and forage legumes growing across a diverse range of environments and geographic regions of the world (e.g., West and South Asia – Pilbeam et al., 1997; Maskey et al., 2001; North and South America – Walley et al., 2007; Espinoza et al., 2012; Africa – Vanlauwe et al., 2019; Oceania – Unkovich et al., 2010; Peoples et al., 2012; Europe – Carlsson and Huss-Danell, 2003; Anglade et al., 2015), management options specifically aimed at supporting greater legume growth will generally have the desired effect of improving inputs of fixed N. The identification of those countries, regions, localities or farming systems with the greatest potential for improvements in legume productivity can be assisted through the judicious use of “yield-gap” analyses, which compare current farmer yields to either experimental or breeders’ plot yields in the same environment, or simulated predictions of “water-limited yield potential” based on climatic records and soil water-holding capacity and nutritional characteristics (Bhatia et al., 2006, Grassini et al., 2015, Van Loon et al., 2018, Tagliapietra et al., 2021). The underlying causes of yield gaps could be further explored using either simulation models and/or meta-analyses of large datasets containing producer field-level yield and management records (Grassini et al., 2015; Hochman et al., 2020; Mourtzinis et al., 2020; Tagliapietra et al., 2021). Often all that might be needed to make progress towards overcoming constraints to productivity revealed by yield-gap analyses is to assist farmers to implement their existing knowledge and adopt known best-management practices (Giller and Cadisch, 1995, Crews and Peoples, 2004). However, in general terms, imposed management strategies would need to consider time of sowing in relation to soil water availability and seasonal water supply, and the length of the effective growing season, as well as avoiding sensitive periods of growth and flowering when there is an elevated probability of frost, drought or high temperatures (Beck et al., 1991, Peoples et al., 2009a, Santachiara et al., 2019, Tagliapietra et al., 2021). Unfavorable and hostile soils which either limit legume root exploration (e.g., soil compaction, sodicity, salinity), inhibit nodulation, or restrict shoot growth (e.g., soil acidity, nutrient deficiencies) should also be ameliorated (Giller and Cadisch, 1995, Peoples et al., 2009a, Santachiara et al., 2019, Vanlauwe et al., 2019, Baijukya et al., 2021). Attention would also need to be given to reducing the incidence of pests, diseases and weed competition responsible for lowering productivity (Beck et al., 1991, Peoples et al., 1995a, Singh et al., 2009; Table 13). In terms of genetic factors, the choice of legume species (and maturity group) most adapted for the local soil type, season or climate is likely to play a crucial role (Peoples et al., 2009a, Tagliapietra et al., 2021), as will plant improvement for enhanced disease resistance (Giller and Cadisch, 1995, Peoples et al., 2019). In the case of forage systems, enhancing legume biomass may require improvements in the proportion, persistence and growth of the legume component of pasture swards through the choice of species mix sown, sowing practices, amelioration of soil acidity, provision of additional phosphorus supply, and management of timing and frequency of grazing or cutting (Ledgard, 2001, Peoples and Baldock, 2001, Carlsson and Huss-Danell, 2003, Rochon et al., 2004, Peoples et al., 2012).

Conceptually, agronomic and breeding objectives designed to enhance the production of BNF by legumes should be accompanied by a consideration of how the net inputs of fixed N provided by legumes in a cropping sequence might be maximized or managed. Such endeavors could utilize simulation models that integrate the effect of climate variability, the retention of the legume and non-legume residues, and changes in the soil water balance and N dynamics and their effects on yield and economic returns from different rotations (Hochman et al., 2020). Simulations models provide the ability to undertake life-cycle analyses of fossil energy consumption, or to design, predict and compare long-term requirements for inputs of N fertilizer and the implicit environmental costs (NO_3_ leaching, greenhouse gas emission outcomes) of different alternative cropping sequences (Costa et al., 2021, Hochman et al., 2021). This would enable the identification of where the N benefits are likely to be the greatest, and provide information on cropping systems and sequences most suited for different agroecological zones (Smith and Chalk, 2020, Tagliapietra et al., 2021). Such approaches can also be utilized as valuable educational tools, to arm producers with the knowledge and information about the likely yield and economic outcomes of different cropping sequence senarios, needed to aid decision-making regarding whether or not to include more legumes in their farming system (Pelzer et al., 2020).

Another factor that would need to be considered is how best to optimize the balance between legumes and non-N_2_-fixing crops. The European study of Ianneta et al. (2016) provided useful insights from this perspective. This applied an annual N balance approach to historical data from eight experimental cropping systems that compared legume and non-legume crop types and systems (e.g., grains, forages and intercrops) across pedoclimatic regions of Europe. The analyses undertaken by Ianneta et al. (2016) revealed that the contribution of BNF to soil N, (a) increased to maximum when the legume fraction was around 0.5 (legume crops present in rotation in half the years), but (b) decreased when the legume fraction increased to 0.6–0.8. The study concluded that inclusion of legumes in rotations have the potential to generate benefits in terms of reducing or dispensing with the need for synthetic N without loss in total output, but that at high frequencies of legumes in a sequence the net inputs of BNF declined. The lower BNF contributions were likely due to a suppression of the N_2_ fixation process due to increased concentrations of soil mineral N, and a build-up of legume pests and pathogens reducing legume growth and vigor.

Perhaps the greatest opportunities and challenges to enhancing the overall BNF contributions by legumes would be achieved by increasing the total area of legumes beyond that currently grown. Inserting additional legumes into cereal-dominated systems also offers an ideal way to meet the desired goals of sustainable crop intensification and diversification (Franke et al., 2018). An increased use of intercropping practices has been proposed as one approach for more legumes to be introduced into the cropping landscape (Lithourgidis et al., 2011, Bedoussac et al., 2015; Fletcher et al., 2016; Vanlauwe et al., 2019). Although the amount of N_2_ fixed per ha tends to be less for intercropped legumes than that of sole crops due to the lower legume productivity, cumulatively each new area of legume grown would be expected to be accompanied by an increase in total global inputs of BNF (Table 13). The enhanced resource use efficiency and higher N acquisition experienced by the intercropped cereal should also result in reductions in the overall N fertilizer required to support cereal production (Jensen et al., 2020). While this is an interesting concept, it might only be suitable for some environments with limited mixes of species and is not without agronomic and logistical challenges.

**Fig. 2:**
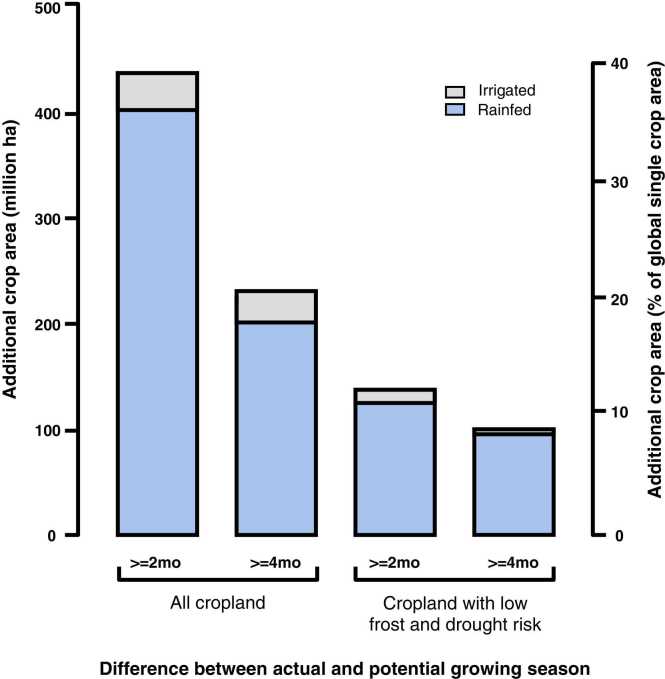
Each bar shows additional crop area measured in hectares and as the percentage of global single cropping area (=1.02 billion ha) under different scenarios, including (a) with a two-or four-month difference between potential and actual growing season, and (b) for all cropland and for cropland with low frost and drought risk (adapted from Waha et al., 2020). Potential for increasing cropping intensity on current global croplands.

Another strategy is to utilize simulation models to identify new arable areas for legume expansion based on the interrogation of climatic and soil type data. A recent global study of cropping systems mapping by Waha et al. (2020) estimated that up to 395 Mha (39% of global single cropping area of 1.02 billion hectares) might potentially accommodate a second crop per year (Fig. 2). This area was largely restricted to rainfed cropping environments. However, after excluding risk prone areas (e.g., frost-occurrence and high rainfall seasonality), the proposed land available for additional cropping where a second growing season of two to four months in duration could be achieved was reduced to between 87 and 131 Mha (9–13% of global single cropping area). The authors acknowledged that these estimates for new areas of double cropping may be overestimated because the cropping intensity in some parts of the world is already higher than the global crop calendar indicates (Waha et al., 2020). The potential for increasing cropping intensity might also be restricted by soil degradation, biotic stresses, as well as lack of input supply, infrastructure, market incentives, processing and storage infrastructure, appropriate technologies or climatic variability (Waha et al., 2020). Nonetheless, some of the designated underutilized area could be dedicated to short duration legume grain crops (e.g., green gram), or used to grow legume forage, green/brown manure or cover crops, all of which would provide some inputs of fixed N, thereby benefitting soil N fertility (Becker et al., 1995, Giller and Cadisch, 1995, Peoples et al., 2017; Table 13). Practical agronomic issues (e.g., the logistical ability of farmers to manage the tight sowing windows immediately after harvesting either the main crop or second crop, plant-back restrictions associated with the application of pesticides, the second crop providing a “green-bridge” which increases disease or insect incidence in subsequent crops) and genetic constraints (e.g. suitably adapted legume germplasm) would both need to be addressed.

Any major expansion of legume area would require the engagement of key players across agriculture value chains and the development of an effective framework to provide elite legume germplasm, appropriate agronomic advisors, stable markets, and possibly new cropping options (Giller and Cadisch, 1995, Fletcher et al., 2016, Franke et al., 2018, Peoples et al., 2019). Perhaps most importantly, the necessary knowledge and right economic incentives would also need to be provided to farmers to give them the confidence to change their current practices and to increase the adoption of legumes in their cropping program (Giller and Ronner, 2019, Peoples et al., 2019, Pelzer et al., 2020).

### 5.2 Future BNF systems for cereals

Transferring N_2_-fixing ability to cereals has been a long-standing goal of plant biologists and has often been referred to as the “holy grail of BNF research” (Hardy and Havelka, 1975, Merrick and Dixon, 1984, Ladha and Reddy, 1995, Triplett, 1996, Beatty and Good, 2011). Many have speculated that if a BNF system could be assembled within the cereal itself, the plant’s internal N demand and N supply could – in theory– be tightly regulated and synchronized. Assembling the BNF trait in crops could also arguably alleviate farmers from the complicated management regimes required to optimize N supply. and greatly reduce the undesirable environmental impacts of the current heavy reliance of cereal production upon synthetic N fertilizer.

In this section, we provide an overview of the different approaches currently being evaluated in the quest to develop functional BNF systems in cereals and other non-legumes. Four major genetic strategies will be described: (a) mucilage-supported BNF (Van Deynze et al., 2018), (b) endophytic diazotrophs that colonize plants to develop nodule-independent N_2_-fixing systems (Ladha and Reddy, 2000), (c) assembling genetic networks for developing root nodule-type symbioses (Ladha and Reddy, 1995, Ladha and Reddy, 2003, Beatty and Good, 2011, Reddy et al., 2013, Rogers and Oldroyd, 2014, Mus et al., 2016), and (d) transferring the genes necessary to assemble an active endogenous nitrogenase enzyme system within the plant (Merrick and Dixon, 1984; Ladha and Reddy, 1995; Curatti and Rubio, 2014; López-Torrejón et al., 2016; Allen et al., 2017; Okada et al., 2020).

#### 5.2.1 Mucilage-supported BNF in cereals

Considerable attention has been focused on an indigenous landrace of maize found in Totontepec Villa de Morelos in the Sierra Mixe region of Mexico, which has highly developed aerial roots that secrete large amounts of carbohydrate-rich mucilage. The mucilage contains a complex mix of polysaccharides (that presumably contribute to its viscosity), and is high in fucose, galactose and arabinose (Van Deynze et al., 2018, Amicucci et al., 2019). Analysis of the mucilage also revealed that it harbors a diverse microbial community which includes many species of diazotrophs (Gonzalez-Ramirez and Ferrera-Cerrato, 1995, Van Deynze et al., 2018). Techniques such as acetylene reduction assay, ^15^N natural abundance, ^15^N dilution, ^15^N_2_-feeding and N balance experiments were applied to demonstrate BNF activity in the mucilage, and to detect contributions of fixed N to maize N nutrition (Van Deynze et al., 2018). The composition of the aerial root mucilage was similar to that secreted from underground roots, and it has been speculated that mucilage-supported diazotrophic communities may also be occurring below-ground (Osborn et al., 1999, Chaboud, 1983).

The idea that mucilage secretion may play a role in harboring diazotrophic microbial communities led to the development of a general model of mucilage-supported BNF (Bennett et al., 2020). Based on the knowledge of the mucilage polysaccharide structure, the proposed model suggested that the diazotrophic microbiota, together with the plant, could fulfill the following four primary functionalities to support N_2_ fixation: (a) disassembly of the complex polysaccharide to release terminal fucose, arabinose and xylose residues, by bacteria and/or plant derived enzymes, (b) utilization of the released fucose, arabinose and/or xylose monosaccharides to fuel microbial nitrogenase activity, (c) reduction of O_2_ tension in the mucilage environment, and (d) lowering of N levels by plant uptake of reduced N from the mucilage. According to published reports, all these functional requirements are present in the Sierra Mixe maize mucilage (Van Deynze et al., 2018, Amicucci et al., 2019, Bennett et al., 2020). Other cereal crops, including conventional maize, sorghum, wheat and barley secrete mucilage, although in much lower quantities, suggesting the possibility that similar mechanisms may already be operative to some degree and that BNF might be stimulated if mucilage secretion could be genetically accentuated.

#### 5.2.2 Enhancing endophytic associations

A wide variety of free-living micro-organisms, including diazotrophs, are found ubiquitously in the rhizosphere of crops and on plant surfaces, and may sometimes enter and survive inside plant tissues as endophytes. Endophytic-bacterial plant associations can be mutualistic to antagonistic and are mostly facultative but can also be present as obligate associations (Nair and Padmavathy, 2014). As far as we know, no diazotrophic obligate association has been reported in any cereals and no diazotrophic endophytes are known to live within healthy plant cells; rather, they are confined to intercellular spaces, the xylem vessels and lignified xylem parenchyma, and dead cells (James, 2000). Reinhold-Hurek and Hurek (2011) reviewed several genomes of endophytes, uncovering attributes associated with rhizosphere competence and gene-encoded products with functions linked to N_2_ fixation. Compared to diazotrophs present on the surface of plants, they found that endophytes colonizing interior plant parts may have a more conducive environment to fix N_2_ and to transfer fixed N to the plant.

Endophytic bacterial associations with cereal plants are typically non-specific, and the density of the bacterial numbers in plant tissues are too low to bolster sufficient N_2_ fixation. Based on the rhizobial numbers in soybean nodules, it was calculated that up to 1 × 10^9^ endophytic bacteria (for example *Azoarcus*) g^-1^ dry weight of plant tissue might be needed for effective N_2_ fixation in rice (see Ladha and Reddy, 2000). Another study estimated a requirement of 5 × 10^8^ cells cm^-3^ per g of fresh weight of wheat roots to achieve significant N_2_ fixation (Katupitiya et al., 1995). Any strategies wishing to engineer endophytic based BNF in cereals would therefore need to develop approaches that enable greater diazotrophic bacterial colonization. Setten et al. (2013) converted a non-diazotrophic but efficient root-colonizing *Pseudomonas protegens* Pf-5 by transferring the *nif* gene assemblage from *Pseudomonas stutzeri* A1501 to create a strain which had the ability to fix N constitutively, even in the presence of combined N. This strain also secreted NH_4_^+^ into the adjoining medium. Subsequent greenhouse experiments showed improved yields in maize and wheat inoculated with this engineered strain, and using ^15^N isotope dilution analysis, demonstrated N_2_ fixation in roots (Fox et al., 2016). A further refinement of *P. protegens* Pf-5 achieved high levels of inducible nitrogenase activity with reduced O_2_ and NH_4_ sensitivity using the *nif* clusters regulated by mutated nifA from *P. stutzeri* and *A. vinelandii* (Ryu et al., 2020). In the modified *P. protegens* Pf-5, nitrogenase activity equivalent to the natural N_2_-fixer was obtained. Ryu et al. (2020) maintained that while this is a good first step towards building efficient strains, additional genetic engineering would be required to (a) maximize the ability of the microorganism to catabolize C sources from the plant, (b) increase the flux of fixed N delivery by redirecting metabolism, and (c) introducing transporters and the optimization of electron transfer. They also proposed the possibility of genetically engineering the plant to produce orthogonal C sources such as opines or less common sugars, and then placing the corresponding catabolism pathways into the bacterium to create a synthetic symbiosis.

#### 5.2.3 Inducing rhizobial symbiosis in cereals

This approach aims to construct a legume-like root nodule symbiotic system in cereals (Reddy et al., 2013, Rogers and Oldroyd, 2014). As a first step towards achieving this, studies were initiated by an international consortium of scientists under the aegis of the global project “Assessing Opportunities for Nitrogen Fixation in Rice” to determine the extent of genetic predisposition of rice for forming symbiosis with rhizobia (see Ladha and Reddy, 2000). The research undertaken in this program as well as subsequent studies demonstrated that many of the genetic programs which aid in the formation of rhizobial symbiosis in legumes are also conserved in rice (see Table 15).

**Table 14:** Comparison of the future potential of different technologies currently being applied to transfer BNF capability to cereals, and the relative benefits and challenges associated with each approach^a^.

Technology	BNF potential	Advantages	Disadvantages	Timeline for delivery	Probability of success
Mucilage supported-BNF	low to medium	The trait is genetically determined by the plant and could be combined with “enhanced” microbes	Mucilage is carbohydrate-rich and may compete with grain for photosynthate	medium-term	low to medium
Endophytic bacterial enhancement	low to medium	Diazotrophs are available now that have enhanced BNF capability, already characterized to some degree	Non-specific, low population of endophytes, poor active transfer of fixed N to plants, difficult to manage, seasonal re-inoculation needed	short- to medium-term	medium
Development of legume-like nodulation by *Rhizobia*	high	Nodulation, a well-known system in legumes, N supply closely synchronized to crop N demand. Seed-based technology	Complex genetic engineering, genetics of plant and bacteria must interact	long-term	low to medium
Nitrogenase expression or transfer to organelle	high	Broad application to crops, N supply would be synchronized to N demand. Seed-based technology	Complex genetic engineering, expression or targeting to chloroplasts or mitochondria	medium- to long-term	medium

a Modified from Ladha and Reddy (1995); Reddy et al. (2002); Bennett et al. (2020).

**Table 15:** Summary of key studies performed for assessing prospects for rice forming legume-like symbioses, and progress made in transfering *nif*^a^ genes to non-diazotrophic hosts including plants.

Highlights	References
**Predisposition of rice for forming N**_**2**_**-fixing symbiosis with rhizobia**	
• Some rice cultivars exude compounds in root exudates that induce transcription of the *nod*^b^ genes of *Rhizobium* species	Reddy et al. (2000); Rolfe et al. (2000)
• Bioengineering of rice plant to produce *nod* gene-inducing flavonoids in roots	Sreevidya et al. (2006)
• Expression of the legume symbiosis-related lectin (*PSL*) and lectin nucleotide phosphohydrolase (*GS52/GsLNP*) genes in rice supported improved intercellular infection/colonization in roots	Sreevidya et al. (2005)
• Evidence for the widespread occurrence of the homologs of early nodulin genes, and common symbiotic pathway genes of legumes in rice	Reddy et al., 1998a, Reddy et al., 1999; Kouchi et al. (1999);Goff et al. (2002); Yu et al. (2002); Gutjahr et al. (2008); Yano et al. (2008); Chen et al., 2007, Chen et al., 2008, Chen et al., 2009; Banba et al. (2008); Markmann and Parniske (2008)
• Demonstration of ability of rice roots to perceive *nod* factors (NF)	Reddy et al., 1998b; Liang et al. (2013); Altúzar-Molina et al. (2020)
• Expression of NF receptor proteins in rice confers root hairs the ability to respond to NFs in terms of exhibiting deformations	
***nif*****gene transfer to non-diazotrophic hosts including plants**	
• Transfer of *nif* gene cluster from N_2_-fixing *Klebsiella pneumoniae*in to *Escherichia coli*	Dixon and Postgate (1972)
• Transfer of iron-only (Anf) nitrogenase system composed of defined *anf* and *nif* genes from *Azotobacter vinelandii* into *Escherichia coli*	Yang et al. (2014)
• Transfer of *Pseudomonas stutzeri* nitrogen fixation island enables expression of active nitrogenase in *Escherichia coli*	Han et al. (2015);Zhang et al. (2015b)
• Expression of refactored *Klebsiella oxytoca*/ *Klebsiella pneumonia nif* gene cluster in *Escherichi acoli*	Temme et al. (2012); Wang et al. (2013); Smanski et al. (2014); Li et al. (2016)
• Transfer of a *nif* cluster from either *Rhodobacter sphaeroides* or *Klebsiella oxytoca* to generate free living N_2_-fixing *Rhizobium* sp IRBG74, and development of ammonium tolerant and oxygen tolerant N_2_-fixing *Pseudomonas protegens* Pf-5 by transferring *nif* cluster from *Pseudomonas stutzeri* and *Azotobacter vinelandii*	Ryu et al. (2020)
• Engineering N_2_ fixation activity in *Synechocystis* 6803 by transferring *nif* gene cluster from *Cyanothece* ATCC 51142 or *Leptolyngbya boryana dg5*	Liu et al. (2018); Tsujimoto et al. (2018)
• Transfer of the *nif* genes from *Klebsiella pneumoniae* to yeast	Zamir et al. (1981); Berman et al., 1985a, Berman et al., 1985b; Holland et al. (1987)
• Generation of active Fe protein by targeting *A. vinelandii* NifH and NifM to mitochondrial matrix, and by expressing NifH, NifM, NifS and NifU in the cytosol of yeast	Lopez-Torrejon et al., 2016
• Formation of nitrogenase NifDK tetramers in the mitochondria of • yeast by targeting *Azotobacter vinelandii* NifH, NifD, NifK, NifU, NifS, NifM, NifE, NifN, and NifB	Burén et al. (2017a)
• Active *Methanocaldococcus infernus* NifB could be produced in yeast mitochondria when co-targetted with *A. vinelandii* NifU, NifS, and FdxN	Burén et al., 2017b, Burén et al., 2019
• Production of FeMo (NifDK) tetramer and the active Fe protein in yeast by simultaneously transforming codon optimized *nifH, nifD, nifK, nifB, nifE, nifN, nifV, nifX, hesA, groES, groEL* of *Pseudomonas polymyxa* WLY78 and *nifF, nifJ, nifS, nifU* of *Klebsiella oxytoca* genes	Liu et al. (2019)
• Biosynthesis of cofactor-activatable iron-only nitrogenase (AnfH) in mitochondrial matrix of yeast	López-Torrejón et al. (2021)
• Identification of superior *Hydrogenobacter thermophilus* NifH protein variant to engineer N_2_ fixation in yeast and plants	Jiang et al. (2021)
• Development of NifD variant for its stable maintenance in mitochondrial matrix of eukaryotic cells (yeast/tobacco)	Allen et al. (2020); Xiang et al. (2020)
• Variations in solubilities of Nif proteins in tobacco mitochondrial environment have been identified (soluble components – NifF, M, N, S, U, W, X, Y and Z; insoluble components – NifB, E, H, J, K, Q and V). The limitations imposed by insolubility of some Nif proteins need to be overcome for successful assembly of nitrogenase in plant mitochondria	Okada et al. (2020)
• Targeting and expressed NifH protein together with NifM into chloroplasts of tobacco could generate functional NifH, although with low activity	Ivleva et al. (2016)
• Production of active nitrogenase Fe protein (NifH) by simultaneously targeting nuclear encoded *Azotobacter vinelandii* NifH, M, U and S components into chloroplasts of tobacco leaf cells	Eseverri et al. (2020)
• Demonstration of the feasibility of targeting and transient expression of the complete range of 16 biosynthetic and catalytic nitrogenase (Nif) proteins in tobacco leaves	Allen et al. (2017)

*In the case of *nif* gene transfer, the studies were ordered according to bacteria, cyanobacteria, yeast and plants.

**All results concerned with *nif* gene expression in plants are obtained with transient expression studies excepting in the investigation conducted by Ivleva et al. (2016), where they were achieved with the plants harboring stably transformed chloroplasts.

^a^*nif* = N_2_ fixation;

^b^nod= nodulation.

Rhizobia readily proliferate in the rhizosphere of rice and can invade roots to colonize intercellular spaces (Reddy et al., 1997), which was shown to be enhanced with the expression of the legume symbiosis-related lectins PSL and GS52 (Sreevidya et al., 2005). Rhizobial nodulation (Nod) factors play a vital role in promoting root hair deformation, infection and root nodule differentiation in legumes. However, in rice roots the rhizobial invasion and colonization process were found to be Nod factor-independent and do not stimulate the development of infection threads (Reddy et al., 1997). Denarie et al. (1996) and Long (1996) highlighted that in legumes, for *Rhizobium* to infect and promote root nodule formation, the bacterial *nod* genes must be induced by plant-produced flavonoids to stimulate the production of Nod factors. Studies in rice also revealed that (a) root exudates of some cultivars could promote *nod* gene induction in *Rhizobium* species (Reddy et al., 2000; Rolfe et al., 2000), and (b) the metabolic pathways in rice roots can be engineered/modified to produce *nod* gene-inducing flavonoids (Sreevidya et al., 2006). More recently, studies have revealed that rice is able to respond to Nod factors and exhibit root hair deformation if rice plants are engineered to express legume-specific Nod factor receptor genes (Altúzar-Molina et al., 2020).

Further studies revealed that rice even has some of the downstream developmental subprograms in its genome that are like those which contribute to nodule development in legumes (Reddy et al., 2002, Ladha and Reddy, 2003). For instance, during the development of nodular symbiosis in legumes, certain plant genes, termed early nodulin (*ENOD*) genes, are induced to promote rhizobial infection and nodule organogenesis. It was shown that rice also possesses homologs of several legume *ENOD* genes including *ENOD40* in its genome (Reddy et al., 1998a, Reddy et al., 1999, Kouchi et al., 1999). Homologs of *ENOD* genes in legumes and rice probably share, at least partially, similar metabolic functions in promoting plant development. For example, evidence has shown that (a) rice and legume *ENOD40*s share analogous roles in formation and/or function of vascular bundles (Kouchi et al., 1999), and (b) the overexpression of *ENOD40*, a critical gene that participates in nodule development in legumes, can trigger cortical cell divisions in rice roots (Reddy et al., 2000, Reddy et al., 2013). With the announcement of the rice genome sequence it became abundantly clear that the homologs of many *ENOD* genes that participate in nodule organogenesis in legumes are conserved to varied degrees in *Oryza* species (Goff et al., 2002, Yu et al., 2002).

**Fig. 3:**
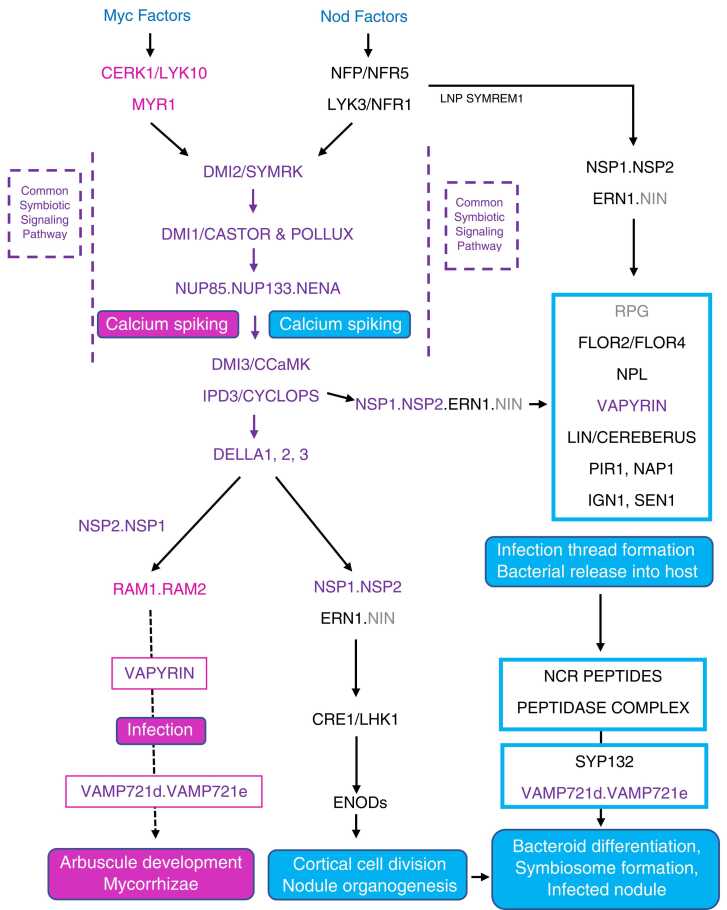
Key genetic elements participating in common symbiotic signaling pathway and specialized cellular processes involved in the development of mycorrhizal (left) and root nodule (right) symbioses (adapted from Reddy et al., 2013).

About 80% of land plants, including legumes and the monocots like rice, can develop endosymbiotic associations with arbuscular mycorrhizal fungi, but only legumes are also capable of recruiting rhizobia for forming nodular symbiotic associations. Several studies demonstrated that in legumes, the genetic components *SYMRK, CASTOR, POLLUX, CCAMK* and *CYCLOPS* (common symbiotic signaling pathway, CSSP) required for nodular symbiosis are also found to be crucial for the establishment of mycorrhizal symbiosis (Fig. 3; see Markmann and Parniske, 2008; Reddy et al., 2013). Interestingly, these same genetic components were found to be central for promoting the formation of endomycorrhizal symbiosis in rice (Gutjahr et al., 2008, Yano et al., 2008, Chen et al., 2007, Chen et al., 2008, Chen et al., 2009, Banba et al., 2008). Transgenic introduction of the rice CSSP gene orthologs into analogous legume mutants was able to promote the development of functional nodules (Yano et al., 2008, Banba et al., 2008, Yokota et al., 2010). This established that the CSSP gene orthologs are functionally conserved in rice, suggesting that many of the components that participate in legume-rhizobial symbiosis are both structurally and functionally conserved in rice. Consequently, they might represent potential buildings blocks for extending genetic networks to accommodate rhizobial symbiosis in rice.

Several critical plant genes encoding the proteins that perceive and transmit Nod signals have been identified in legumes, and substantial progress has been made in defining the initial signaling networks (i.e. CSSP) which play a vital part in promoting rhizobial infection and nodule organogenesis (see Oldroyd, 2013). Integration of these legume genes with the existing CSSP of non-leguminous plants may enable them to perceive and respond to rhizobial nodulation signals. It is notable that ectopic expression of legume-specific Nod factor receptors in rice enabled root hairs to respond to Nod factors by means of exhibiting deformations like in legumes (Altúzar-Molina et al., 2020). In summary, (a) the recent advances in our knowledge on legume-rhizobia symbioses, and (b) discovery of a “common symbiotic signaling pathway” in the plants forming arbuscular mycorrhizal and rhizobial symbioses provide a basis by which to explore the potential for extending the mycorrhizal symbiotic genetic network of non-legumes to accommodate rhizobial symbiosis (Markmann and Parniske, 2008, Reddy et al., 2013, Rogers and Oldroyd, 2014).

Exploratory studies described in the preceding paragraphs suggest that it may be possible to engineer cereals to accommodate symbiosis with N_2_-fixing rhizobia. However, besides the bioengineering tasks, there are two other challenges needing to be addressed in order to achieve a fully functional N_2_-fixing symbiosis in cereals. Firstly, the ATP for N_2_ fixation by rhizobia comes from respiration, which requires O_2_. In legume nodules, N_2_ fixation requires strict regulation of O_2_ supply to the rhizobial cells, because although O_2_ is needed for respiration, O_2_ concentrations well below atmospheric levels can irreversibly damage the nitrogenase enzyme. To circumvent this problem, legumes have evolved variable physiologically-controlled physical diffusion barriers, which maintain a low O_2_ concentration around nitrogenase while providing a high O_2_ flux, supporting high respiration rates by the bacteroids (Deninson and Kinraide, 1995; Wei and Layzell, 2006). A high respiratory utilization of O_2_ and a cortical impediment to its diffusion were suggested to work in tandem to maintain a low, non-inhibitory level of O_2_ concentration at the central infection zone in order to promote N_2_ fixation in legume nodules (Layzell and Hunt, 1990). Cereals hosting rhizobia would need somewhat analogous system(s) for supporting BNF at an affordable C cost.

Secondly, legumes have evolved mechanisms which selectively allocate resources to the most beneficial rhizobia to support their growth in nodules and impose sanctions on less or non-beneficial rhizobia in order to prevent a drain on plant resources due to their colonization of the host tissues (Denison, 2000). Production of photosynthates as well as the light-induced signals generated in the leaves trigger nodulation in roots (Wang et al., 2021). There is evidence that in nodules, C allocation by the host plant also acts as a sanctioning tool which differentially controls growth of inefficient and efficient N_2_-fixing rhizobial strains – low N_2_-fixing rhizobia received less C when they shared the host with high-fixing rhizobial strains (Westhoek et al., 2021). In legume nodules, decreased O_2_ supply may precede decreased C supply (Kiers et al., 2003, Denison, 2021). Similar mechanisms would need to be developed for cereals. In the absence of host-imposed sanctions against less-beneficial strains, the net benefits derived from any BNF activity would be negligible (West et al., 2002). Hence, physiological regulation of O_2_ permeability in cereal roots may be an effective mechanism for limiting wasteful resource use by less-beneficial rhizobia (Kiers et al., 2003).

#### 5.2.4 *nif* gene transfer

This strategy involves embedding the genetic pathways of bacterial nitrogenase directly into the plant genome, and assembling and functioning the translated proteins within the plant cell. About 20 N_2_ fixation *nif* genes participate in the biosynthesis and nitrogenase activity in diazotrophic bacteria. Genes essential for BNF can be classified into three functional groups: (a) those encoding electron-transport components, (b) proteins essential for metal cluster biosynthesis, and (c) the main nitrogenase apoenzyme (catalytic components) (Yang et al., 2018). The apparent complexity of nitrogenase biosynthesis, the O_2_ sensitivity of nitrogenase enzyme, and high requirement for energy (ATP) and reducing power (NADPH) are major impediments for introducing such N_2_ fixation traits into plants.

In a breakthrough investigation, Dixon and Postgate (1972) transferred *nif* genes from *Klebsiella pneumoniae* to *Escherichia coli*, converting this non-fixing bacterium into one capable of growing in the absence of combined N. This gave plant biologists hope that the attribute could also be transmitted to more complex organisms such as plants. Soon after this discovery, Hardy and Havelka (1975) envisioned new technologies to generate crops which could synthesize their own fixed N. Since this period, however, significant additional progress has been made in *nif* gene transfer to non-diazotrophic hosts including plants (Table 15).

Since BNF requires large amounts of ATP and NADPH, mitochondria (Curatti and Rubio, 2014) and chloroplasts (Merrick and Dixon, 1984) have both been suggested as appropriate locations to establish a N_2_-fixing apparatus. To avoid O_2_ produced during photosynthesis in chloroplasts damaging to the integrity of the nitrogenase complex it was proposed that nitrogenase expression in chloroplasts needed to separate BNF and photosynthesis temporally or spatially by either temporally restricting nitrogenase expression only during the night, or by spatially limiting *nif* gene expression to roots. Curatti and Rubio (2014) considered localization of nitrogenase in the mitochondrial matrix as being more conducive for the nitrogenase enzyme because of the near-O_2_-free environment generated inside the mitochondria as a result of high respiratory activity.

Based on the genetic transformation of *nif* genes in *E. coli*, it is believed that a minimum set of 12–14 (*nifH, nifD, nifK, nifB, nifE, nifN*, *nifU, nifS, nifJ, nifV, nifW, nifF, nifM* and *nifY*) of the many *nif* genes would be sufficient for the biosynthesis of the core nitrogenase apoenzyme and to maintain its activity in vivo (Rubio and Ludden, 2008, Curatti and Rubio, 2014, Burén et al., 2019), because the products of other genes that are essential for proteins required for metal cluster biosynthesis in vivo could be complemented by the activities of plant counterparts operating in mitochondria or chloroplasts (Curatti and Rubio, 2014, Yang et al., 2018). As part of a Bill and Melinda Gates Foundation project, various strategies are being evaluated to assemble active nitrogenase enzyme in eukaryotic organisms using yeast as a model system (see Burén et.al, 2017a). López-Torrejón et al. (2016) expressed *A. vinelandii nifH, nifM, nifS* and *nifU* genes in yeast and demonstrated that active nitrogenase reductase (Fe-protein, encoded by *nifH*) can be generated if the NifH polypeptide is targeted to the mitochondrial matrix, together with the NifM maturase. López-Torrejón et al. (2016) further showed that to produce a functional Fe protein in yeast, simultaneous transfer of Nif-specific Fe–S cluster biosynthetic proteins NifU and NifS into mitochondrial matrix was not required, as NifH was able to acquire/incorporate endogenously-generated mitochondrial Fe–S clusters. In a following study with yeast, Burén et al. (2017b) targeted a minimum set of nine *A. vinelandii nif* gene products (NifH, NifD, NifK, NifM, NifB, NifU, NifS, NifE and NifN) into the mitochondrial matrix and demonstrated the formation of potential NifDK tetramer, a crucial first step in assembling a functional nitrogenase in a eukaryotic cell.

Through transient gene expression studies in tobacco leaves, Allen et al. (2017) showed that the full array of both biosynthetic and catalytic nitrogenase proteins from *Klebsiella pneumoniae* can be individually expressed as mitochondrial-targeting peptide-Nif fusions. They transiently expressed 16 Nif proteins of *K. pneumoniae* in tobacco and targeted to the mitochondria, but none of the Fe and MoFe proteins showed activity. Okada et al. (2020) found that among mitochondria-targeted Nif proteins in tobacco chloroplasts, only NifM, NifU, NifS, NifN, NifF, NifX, NifY, NifZ and NifW were in soluble form while NifH, NifK, NifB, NifE, NifJ, NifQ and NifV were insoluble. Another set of studies demonstrated that *A. vinelandii* NifH (Eseverri et al., 2020) and NifB (Burén et al., 2017a) proteins were sequestered as insoluble forms in tobacco chloroplasts. Concomitant investigation with NifH protein suggests that it can be brought into soluble form if co-localized in chloroplasts with NifM protein (Eseverri et al., 2020). To make progress in the development of N_2_-fixing plants, insoluble forms of other Nif proteins will need to be similarly rectified.

Another challenge needing to be overcome relates to the NifD protein. Studies in yeast (Burén et al., 2017a) and tobacco (Allen et al., 2017) both indicated that the NifD polypeptide is susceptible to degradation in the mitochondrial matrix of eukaryotic cells, thus affirming a requirement for optimizing its polypeptide sequence to enhance stability without compromising catalytic activity. Fortunately, two research groups using synthetic biology have recently generated NifD protein variants capable of resisting mitochondrial degradation in yeast (Allen et al., 2020, Xiang et al., 2020), tobacco and *Arabidopsis* (Allen et al., 2020).

Less effort has been directed towards *nif* gene transfer to chloroplasts of photosynthetic eucaryotes, although preliminary attempts at *nif* gene transfer were undertaken in algae and plants. Cheng et al. (2005) expressed *K. pneumoniae* nifH in *Chlamydomonas reinhardii* and showed that it could substitute for chlL, a gene essential for chlorophyll biosynthesis, thereby providing evidence for *NifH* functionality in this green alga. Utilizing chloroplast transformation technology, Ivleva et al. (2016) expressed *A. vinelandii* NifH protein together with NifM in chloroplasts of tobacco plants which produced functional NifH, albeit with low activity. Recently, using transient expression assays in tobacco leaves, Eseverri et al. (2020) showed that for functional constitution of NifH in chloroplasts, NifU and NifS also need to be simultaneously targeted, along with NifM for generation of active NifH in plant chloroplast matrix.

#### 5.2.5 Will energy costs incurred by *in planta* N_2_ fixation impair cereal productivity?

The N_2_ fixation process requires large amounts of metabolic energy, and there have been concerns that transmuting non-N_2_-fixing cereals into N_2_-fixing species would penalize their productivity if the induced BNF in cereals represented a large alternative sink for photosynthates (Rosenblueth et al., 2018). We can assess this risk by comparing the energy costs of N acquisition by legumes from either NO_3_^-^ uptake or N_2_ fixation. When N is acquired from NO_3_^-^ assimilation, first NO_3_^-^ needs to be converted to NH_4_^+^ and then synthesized into amino acids – depending upon legume species, this occurs predominantly in the roots or leaves (Pate, 1980). In case of N_2_-fixing legumes, N_2_ is first reduced to NH_4_^+^ prior to supporting the enzymatic transformation into more complex forms of N such as ureides, amides and amino acids (depending upon species) to be exported from the nodule in the xylem stream (Pate, 1980, Herridge and Peoples, 2002). Energy budgets and respiratory requirements for the conversion of NO_3_^-^ or N_2_ into NH_4_^+^ have been calculated for several legume species (Pate et al., 1979, Kennedy and Cocking, 1997, Jensen et al., 2012). It was concluded that there would be higher respiratory losses to support BNF with the C/energy expended for the transition of N_2_ to NH_4_^+^ by the nitrogenase system estimated to be ΔG = −687 kJ mol^-1^, compared to a C/energy budget for the transformation of NO_3_^-^ to NH_4_^+^ of around ΔG = −605 kJ mol^-1^. Due to this slightly higher energy requirement for N_2_ fixation, it was speculated that the additional C costs of BNF compared to soil sources of N would result in lower amounts of C being allocated for above-ground growth by legumes reliant upon BNF for growth (Vance and Heichel, 1991). However, no conclusive or consistent experimental evidence has corroborated this hypothesis. Some greenhouse experiments detected lower legume biomass when plants were grown using N_2_ versus NO_3_^-^ (Gibson, 1966, Xie et al., 2015), but either no or only minor differences in yield were observed under field conditions (Rigaud, 1981, Salles de Oliveira et al., 2004, Reinprecht et al., 2020). These studies suggest that the consequences of changing cereal reliance from NO_3_^-^ to BNF might be small.

The energy load for N assimilation in plants tends to be lower when NH_4_^+^ is provided as an N source. Consequently, ammonium sulfate and urea are routinely used to fertilize cereals like rice. In some rice cultivars, under lowland flooded conditions, N uptake and plant growth can be improved if NO_3_^-^ is supplied along with NH_4_^+^ (Xiaoe and Xi, 1991, Ancheng et al., 1993, Glass and Siddiqi, 1995, Kronzucker et al., 1999, Kronzucker et al., 2000). This suggests that rice can provide the sufficient additional resources to facilitate the assimilation of NO_3_^-^ without compromising yield, and that energy supply is not limiting or it can be up-regulated to compensate for increased demand. Certainly, there is evidence in soybean that photosynthesis is stimulated by C sink strength created by rhizobial as well as mycorrhizal symbiosis (Kaschuk et al., 2009), and that photosynthetic rates can be higher when soybean is dependent upon BNF for growth than when reliant upon NO_3_^-^ (Kaschuk et al., 2010). It is well known that the balance between photosynthesis-mediated sugar production in the chloroplast-harboring leaf cells (source tissues) and carbohydrate consumption by roots, shoots and grains (sink tissues) must be preserved to support plant development and growth. Under ideal light conditions and at ambient CO_2_ levels, sink restraint ensues when the rate of photosynthesis is restricted by inadequate purging of photosynthetic products produced in green plant tissues through the Calvin–Benson cycle (Sawada et al., 1986, Sharkey et al., 1986, Paul and Foyer, 2001, Adams et al., 2013).

Since cereal biomass has lower N content, and grains have lower concentrations of protein than protein-rich legumes, it has been speculated that lower levels of BNF might be required by N_2_-fixing cereals to satisfy their growth requirements, resulting in a smaller photosynthetic demand than an equivalent system in symbiotic legumes (Ladha and Reddy, 1995). It has been estimated that as much as 29% of photosynthate is exuded into the soil surrounding cereal roots (Vives-Peris et al., 2019), so cereals might already have the capacity to tolerate the additional energy costs of BNF without greatly compromising their yield. If needed, some of the C sources currently released in root exudates could perhaps be diverted to support N_2_ fixation, although in this case viable mechanisms for limiting root exudation would need to be explored.

## 6 Summary and conclusions

Given that N supply is frequently the second most limiting factor after water availability constraining crop growth, it is no surprise that farmer demand for convenient sources of N, such as fertilizer, increased once new cereal varieties with higher genetic yield potential started to be released after the “green revolution” in the middle of the twentieth century. The on-going demand for N fertilizer has continued to grow, driven largely by the progressive improvements in cereal production needed to feed the human population, as it grew from around 3 billion in 1960 to 7.9 billion by 2021, so that the amount of synthetic N now applied to wheat, rice and maize represents > 50% of the total fertilizer consumed globally by agriculture. In the absence of any significant changes in dietary habits, it has been estimated that a further two- to three-fold increase in N supply will be required to support global food production to satisfy the requirements of the anticipated population of 9.7 billion by the second half of the twenty-first century. There are clear implications of escalating environmental damage if fertilizers synthesized by the Haber-Bosch process if this remains the primary source of N used to satisfy this increased demand. While efforts need to continue to improve the NUE of fertilizer N and to lower the undesirable environmental impact arising from N loss processes, it is our proposition that since the dominant sources of BNF provide improved environmental outcomes compared to cropping systems reliant upon fertilizer N, BNF should play a larger role in supporting the future projected growth in cereal production. We also believe that greater attention and well targeted research will be required to enhance the purposeful use of BNF.

Biological N_2_ fixation is a key process in the global N cycle and is an important mechanism for replenishing the soil reservoirs of organic N and improving the soil’s ability to supply plant-available forms of N for crop uptake. Although a relatively limited number of bacterial and archaeal species fix N_2_, they represent a wide variety of phylogenetically and physiologically distinct types that occupy different niches. It was estimated that in 2019, global inputs of BNF in cereal-based cropping systems derived from grain legumes (34.4 Tg N) and non-symbiotic sources (15.6 Tg N) represented 50 Tg of fixed N. The review described a range of opportunities where inputs of BNF could potentially be increased beyond what is currently being achieved.

The identification of effective strategies to raise non-symbiotic BNF inputs is challenging because of the loose association of N_2_-fixing bacteria in cereal cropping systems. However, the use of genetic tools to investigate the influence of agronomic management and crop genotype on the abundance of N_2_-fixing diazotrophs in the root-soil micobiome holds some hope of assessing new and novel ways of manipulating BNF inputs. Early results suggest greater adoption of cropping systems managed with reduced soil disturbance, and the maintenance of crop residues as standing stubble of mulches, to be promising entry points for increasing BNF by free-living diazotrophs. Higher non-symbiotic BNF has been reported to occur with diazotroph inoculation and genotypic differences in cereal host cultivar, although further research is required to consistently demonstrate these findings under field conditions.

Many plant biologists have speculated that the ultimate solution for solving the ever-growing N challenge is to bestow cereals (and other economically important crops) with their own capacity for BNF. Our review suggests that recent breakthroughs in the genomics of diazotrophs and the genetics of BNF, as well as improvements in the understanding of the processes involved in legume-rhizobia symbioses, have opened up new avenues by which to tackle this problem much more systematically. Likewise, advances in the field of *nif* gene expression in eukarotic systems offer another means of possibly achieving N_2_-fixing cereal crops. Nonetheless, all the approaches under evaluation are extremely high-risk scientific endeavors and the transfer of BNF capacity to cereals remains a long-term and uncertain goal. If, however, N_2_-fixing cereals should ultimately be achieved, the deployment of the technology would need to be carefully managed to avoid the undesirable reduction in cropping diversification that would accompany an expansion of cereal monoculture systems.

Aquatic green manure crops (*Azolla* and legumes) can fix considerable amounts of N_2_, but in modern lowlands rice-based farming systems their utilization is greatly limited by technological, environmental and socioeconomic imperatives, and these sources of fixed are therefore unlikely to be major contributors in future intensive cropping. It was concluded that agronomic management and appropriate improved legume germplasm to address current yield gaps and raise legume productivity, combined with an expansion in the area of grain, forage, green manure and cover-crop legumes grown in cereal-based cropping systems, represent the strongest prospects for enhancing total inputs of fixed N and supporting the soil’s ability to supply cereals with more plant-available N. As a vehicle for diversification, the more frequent inclusion of legumes in cropping sequences would also assist the long-term resilience of otherwise cereal-dominated farming systems, reduce the fossil energy C costs of food production, and lower net green-house gas emissions.

The review highlights the ways in which BNF will need to be a core component of efforts to build more sustainable agroecosystems. To be both increasingly productive and sustainable, future cereal cropping systems will need to better incorporate and leverage natural processes, such as BNF, to reduce the inefficiencies and externalities associated with excessive synthetic N use.

## Declaration of Competing Interest

The authors declare that they have no known competing financial interests or personal relationships that could have appeared to influence the work reported in this paper.
