# Supplementary material for: Biological nitrogen fixation and prospects for ecological intensification in cereal-based cropping systems
Source: Field Crops Res. 2022 Jul 1;283:108541. doi: 10.1016/j.fcr.2022.108541 (PMC9133800; doi:10.1016/j.fcr.2022.108541)
Supplement: Table S1 — Supplementary material [file mmc1.docx]

**Ms. No. AGEE31437**

**Title：**Coupling side-deep fertilization with Azolla to reduce ammonia volatilization while achieving a higher net economic benefits in rice cropping system

**Names of authors:** Wen-Bin Zhu ^a,b^, Ke Zeng ^a,b^, Yu-Hua Tian ^a,c^, Bin Yin ^a, *^

**Complete postal address(es) or affiliations:**

^a^ State Key Laboratory of Soil and Sustainable Agriculture, Institute of Soil Science, Chinese Academy of Sciences, Nanjing 210008, China

^b^ University of Chinese Academy of Sciences, Beijing 100049, China

^c^ Changshu National Agro-Ecosystem Observation and Research Station

**Full telephone, Fax No. and E-mail address of the corresponding author:**

**^*^Corresponding author:** Bin Yin

**Tel.:** +86 25 86881024**, Fax:** +86 25 86881000

**E-mail:** [byin@issas.ac.cn](mailto:byin@issas.ac.cn)

**Supplementary material:**

**Table S1** Variability of model input parameters for Monte Carlo simulation

|  | CK | CN | RN | RNS | RNSA |
| --- | --- | --- | --- | --- | --- |
| Measurement index | | | | | |
| Rice yield  (kg ha^-1)^ | 4400.14± 600.31 | 9315.90± 248.79 | 8855.81± 376.72 | 9299.42± 526.51 | 9766.14± 242.61 |
| NH_3_ emissions  (kg N ha^-1^) | 11.11± 1.31 | 50.89± 7.62 | 42.65± 7.35 | 29.47± 4.60 | 23.63± 5.72 |
| Price type | | | | | |
| P_A_ (yuan kg^-1^) | 4.50-6.00 | | | | |
| P_E_ | 3.87-4.88 | | | | |
| P_D_ | 2.52-20.79 | | | | |
| P_R_ | 2.28-3.28 | | | | |
| P_N_ | 3.80-4.50 | | | | |
| P_P_ | 2.00-3.20 | | | | |
| P_K_ | 3.20-4.00 | | | | |
| P_SR_ (yuan ha^1^) | 1650.00-1800.00 | | | | |
| P_F_ | 800.00-1500.00 | | | | |
| P_G_ | 937.50-1125.00 | | | | |

P_A,_ P_E_, and P_D_ : the cost per kg of SO_2_ equivalent in acidification, eutrophication, and health damage; P_R_: the price of rice grain; P_N_, P_P_, and P_K_: the price of nitrogen, phosphorus, and potassium; P_SR_: the price of rice seeds; P_F_ and P_G_: the cost of fertilization input and green manure.

**Table S2** Uncertainties of NEB in different fertilization treatments

| Treatments | Maximum | Minimum | Mean | Std. deviation | CV |
| --- | --- | --- | --- | --- | --- |
|  | 10^3^ CNY ha^-1^ | | | | % |
| CK | 16.25 | 2.38 | 8.46 | 2.11 | 24.93 |
| CN | 26.67 | 13.65 | 19.98 | 2.77 | 13.86 |
| RN | 27.23 | 12.49 | 19.05 | 2.76 | 14.46 |
| RNS | 29.61 | 12.17 | 20.58 | 3.05 | 14.83 |
| RNSA | 30.57 | 14.03 | 20.92 | 2.87 | 13.68 |

CV: coefficient of variation.

**Fig. S1.** Mean daily air temperature and precipitation in 2019 and 2020.
